# Supplementary material for: Risk factors and transmission pathways associated with infant Campylobacter spp. prevalence and malnutrition: A formative study in rural Ethiopia
Source: PLoS One. 2020 May 8;15(5):e0232541. doi: 10.1371/journal.pone.0232541 (PMC7209302; doi:10.1371/journal.pone.0232541)
Supplement: Attachment — Submitted filename: revised-PONE-D-19-33566_R1_reviewer.pdf [file pone.0232541.s005.pdf]

PLOS ONE

Risk factors and transmission pathways associated with infant Campylobacter spp. prevalence and malnutrition: A formative study in rural Ethiopia

--Manuscript Draft--

**Manuscript Number:**	PONE-D-19-33566R1
**Article Type:**	Research Article
**Full Title:**	Risk factors and transmission pathways associated with infant Campylobacter spp. prevalence and malnutrition: A formative study in rural Ethiopia
**Short Title:**	Infant Campylobacter prevalence, malnutrition and domestic animal ownership in rural Ethiopia
**Corresponding Author:**	Alison Parker<br>Cranfield University<br>Cranfield, UNITED KINGDOM
**Keywords:**	Campylobacter; poultry; malnutrition, Ethiopia
**Abstract:**	Early infection from enteropathogens is recognised as both a cause and effect of infant malnutrition . Specifically, evidence demonstrates associations between growth shortfalls and Campylobacter infection, endemic across low-income settings, with poultry a major source. Whilst improvements in water, sanitation and hygiene (WASH) should reduce pathogen transmission, interventions show inconsistent effects on infant health. This cross-sectional, formative study aimed to understand relationships between infant Campylobacter prevalence, malnutrition and associated risk factors, including domestic animal husbandry practices, in rural Ethiopia. Thirty-five households were visited in Sidama zone, Southern Nations, Nationalities and Peoples' region. Infant and poultry faeces and domestic floor surfaces (N=102) were analysed for presumptive Campylobacter spp. using selective culture. Infant anthropometry and diarrhoeal prevalence, WASH facilities and animal husbandry data were collected. 14.3% of infants were wasted, 31.4% stunted and 31.4% had recent diarrhoea. Presumptive Campylobacter spp. was isolated from 48.6% of infant, 68.6% of poultry and 65.6% of floor surface samples . Compared to non-wasted infants, wasted infants had an increased odds ratio (OR) of 1.41 for a Campylobacter -positive stool and 1.81 for diarrhoea. Positive infant stools showed a significant relationship with wasting (p=0.026) but not stunting. Significant risk factors for a positive stool included keeping animals inside (p=0.027, OR 3.5), owning cattle (p=0.018, OR 6.5) and positive poultry faeces (p<0.001, OR 1.34). Positive floor samples showed a significant correlation with positive infant (p=0.023), and positive poultry (p=0.013, OR 2.68) stools. Ownership of improved WASH facilities was not correlated with lower odds of positive stools. This formative study shows a high prevalence of infants positive for Campylobacter in households with free-range animals. Findings reaffirm contaminated floors as an important pathway to infant pathogen ingestion and suggest that simply upgrading household WASH facilities will not reduce infection without addressing the burden of contamination from animals, alongside adequate separation in the home.
**Order of Authors:**	Alison Parker
	Sophie Budge
	Megan Barnett
	Paul Hutchings
	Sean Tyrrel
	Francis Hassard
	Camila Garbutt
	Mathewos Moges
	Fitsume Woldemedhin
	Mohammedyasin Jemal

Powered by Editorial Manager® and ProduXion Manager® from Aries Systems Corporation

Response to Reviewers:	See detailed response attached.
Additional Information:	
Question	Response
**Financial Disclosure**<br><br>Enter a financial disclosure statement that describes the sources of funding for the work included in this submission. Review the submission guidelines for detailed requirements. View published research articles from *PLOS ONE* for specific examples.<br><br>This statement is required for submission and **will appear in the published article** if the submission is accepted. Please make sure it is accurate.<br><br>**Unfunded studies**<br>Enter: *The author(s) received no specific funding for this work.*<br><br>**Funded studies**<br>Enter a statement with the following details:<br>• Initials of the authors who received each award<br>• Grant numbers awarded to each author<br>• The full name of each funder<br>• URL of each funder website<br>• Did the sponsors or funders play any role in the study design, data collection and analysis, decision to publish, or preparation of the manuscript?<br>• **NO** - Include this sentence at the end of your statement: *The funders had no role in study design, data collection and analysis, decision to publish, or preparation of the manuscript.*<br>• **YES** - Specify the role(s) played.<br><br>* typeset	SB is partly funded as a research student by People in Need. https://www.clovekvtisni.cz/en/ CG, FW and MJ from People in Need adviced on the practicalities of study design, helped with data collection and reviewed the manuscript.
**Competing Interests**<br><br>Use the instructions below to enter a competing interest statement for this submission. On behalf of all authors, disclose any competing interests that could be perceived to bias this work—acknowledging all financial support and any other relevant financial or non-financial competing interests.	The authors have declared that no competing interests exist.

Powered by Editorial Manager® and ProduXion Manager® from Aries Systems Corporation

This statement **will appear in the published article** if the submission is accepted. Please make sure it is accurate. View published research articles from *PLOS ONE* for specific examples.<br><br>**NO authors have competing interests**<br><br>Enter: *The authors have declared that no competing interests exist.*<br><br>**Authors with competing interests**<br><br>Enter competing interest details beginning with this statement:<br><br>*I have read the journal's policy and the authors of this manuscript have the following competing interests: [insert competing interests here]*<br><br>* typeset	
**Ethics Statement**<br><br>Enter an ethics statement for this submission. This statement is required if the study involved:<br><br>• Human participants<br>• Human specimens or tissue<br>• Vertebrate animals or cephalopods<br>• Vertebrate embryos or tissues<br>• Field research<br><br>Write "N/A" if the submission does not require an ethics statement.<br><br>General guidance is provided below. Consult the submission guidelines for detailed instructions. **Make sure that all information entered here is included in the Methods section of the manuscript.**	Cranfield University Research Ethics Committee (CURES/7774/2019)<br>Hawassa University College of Medicine and Health Sciences (IRB/222/11).<br>Oral consent was obtained from adult parents/guardians of child participants. There were no adult participants. Oral consent was obtained because most caregivers were illiterate; this was approved by both IRBs. It was recorded by writing on the survey form.

**Format for specific study types**<br><br>**Human Subject Research (involving human participants and/or tissue)**<br>• Give the name of the institutional review board or ethics committee that approved the study<br>• Include the approval number and/or a statement indicating approval of this research<br>• Indicate the form of consent obtained (written/oral) or the reason that consent was not obtained (e.g. the data were analyzed anonymously)<br><br>**Animal Research (involving vertebrate animals, embryos or tissues)**<br>• Provide the name of the Institutional Animal Care and Use Committee (IACUC) or other relevant ethics board that reviewed the study protocol, and indicate whether they approved this research or granted a formal waiver of ethical approval<br>• Include an approval number if one was obtained<br>• If the study involved *non-human primates*, add *additional details* about animal welfare and steps taken to ameliorate suffering<br>• If anesthesia, euthanasia, or any kind of animal sacrifice is part of the study, include briefly which substances and/or methods were applied<br><br>**Field Research**<br><br>Include the following details if this study involves the collection of plant, animal, or other materials from a natural setting:<br>• Field permit number<br>• Name of the institution or relevant body that granted permission	
**Data Availability**<br><br>Authors are required to make all data underlying the findings described fully available, without restriction, and from the time of publication. PLOS allows rare exceptions to address legal and ethical concerns. See the PLOS Data Policy and FAQ for detailed information.	Yes - all data are fully available without restriction

A Data Availability Statement describing where the data can be found is required at submission. Your answers to this question constitute the Data Availability Statement and **will be published in the article**, if accepted.<br><br>**Important:** Stating 'data available on request from the author' is not sufficient. If your data are only available upon request, select 'No' for the first question and explain your exceptional situation in the text box.<br><br>Do the authors confirm that all data underlying the findings described in their manuscript are fully available without restriction?	
**Describe where the data may be found in full sentences. If you are copying our sample text, replace any instances of XXX with the appropriate details.**<br><br>• If the data are **held or will be held in a public repository**, include URLs, accession numbers or DOIs. If this information will only be available after acceptance, indicate this by ticking the box below. For example: *All XXX files are available from the XXX database (accession number(s) XXX, XXX.).*<br>• If the data are all contained **within the manuscript and/or Supporting Information files**, enter the following: *All relevant data are within the manuscript and its Supporting Information files.*<br>• If neither of these applies but you are able to provide **details of access elsewhere**, with or without limitations, please do so. For example:<br><br>*Data cannot be shared publicly because of [XXX]. Data are available from the XXX Institutional Data Access / Ethics Committee (contact via XXX) for researchers who meet the criteria for access to confidential data.*<br><br>*The data underlying the results presented in the study are available from (include the name of the third party*	All data created during this research will be openly available from the Cranfield Online Research Data at 10.17862/cranfield.rd.9907385.

and contact information or URL).<br>• This text is appropriate if the data are owned by a third party and authors do not have permission to share the data.<br><br>* typeset	
Additional data availability information:	Tick here if the URLs/accession numbers/DOIs will be available only after acceptance of the manuscript for publication so that we can ensure their inclusion before publication.

Powered by Editorial Manager® and ProduXion Manager® from Aries Systems Corporation

Clean Manuscript
Click here to access/download;Manuscript;S Budge et al_2020_Revised manuscript clean.docx

Risk factors and transmission pathways associated with infant *Campylobacter spp.* prevalence and malnutrition: A formative study in rural Ethiopia

Short title

Infant *Campylobacter* prevalence, malnutrition and domestic animal ownership in rural Ethiopia

Sophie Budge[a¶], Megan Barnett[b¶], Paul Hutchings,[a¶] Alison Parker[a*¶],, Sean Tyrrel[a&], Francis Hassard[a&], Camila Garbutt[c&], Mathewos Moges[d&] Fitsume Woldemedhin[e], Mohammedyasin Jemal[e]

[a] *Cranfield University, College Road, Cranfield, UK.*

[b] *British Geological Survey, Environmental Science Centre, Keyworth, Nottingham, UK.*

[c] *People in Need UK, 49-51 East Road, London, UK.*

[d] *Hawassa University College of Medicine and Health Sciences, Department of Environmental Health, Hawassa, Ethiopia*

[e] *People In Need, Hawassa office, Ethiopia.*

***** Corresponding author

Email: a.parker@cranfield.ac.uk (AP).

¶ These authors contributed equally to this work.

& These authors also contributed equally to this work.

Abstract

Early infection from enteropathogens is recognised as both a cause and effect of infant malnutrition. Specifically, evidence demonstrates associations between growth shortfalls and *Campylobacter* infection, endemic across low-income settings, with poultry a major source. Whilst improvements in water, sanitation and hygiene (WASH) should reduce pathogen transmission, interventions show inconsistent effects on infant health. This cross-sectional, formative study aimed to understand relationships between infant *Campylobacter* prevalence, malnutrition and associated risk factors, including domestic animal husbandry practices, in rural Ethiopia. Thirty-five households were visited in Sidama zone, Southern Nations, Nationalities and Peoples' region. Infant and poultry faeces and domestic floor surfaces (N=102) were analysed for presumptive *Campylobacter* spp. using selective culture. Infant anthropometry and diarrhoeal prevalence, WASH facilities and animal husbandry data were collected. 14.3% of infants were wasted, 31.4% stunted and 31.4% had recent diarrhoea. Presumptive *Campylobacter* spp. was isolated from 48.6% of infant, 68.6% of poultry and 65.6% of floor surface samples. Compared to non-wasted infants, wasted infants had an increased odds ratio (OR) of 1.41 for a *Campylobacter*-positive stool and 1.81 for diarrhoea. Positive infant stools showed a significant relationship with wasting (p=0.026) but not stunting. Significant risk factors for a positive stool included keeping animals inside (p=0.027, OR 3.5), owning cattle (p=0.018, OR 6.5) and positive poultry faeces (p<0.001, OR 1.34). Positive floor samples showed a significant correlation with positive infant (p=0.023), and positive poultry (p=0.013, OR 2.68) stools. Ownership of improved WASH facilities was not correlated with lower odds of positive stools. This formative study shows a high prevalence of infants positive for *Campylobacter* in households with free-range animals. Findings reaffirm contaminated floors as an important pathway to infant pathogen ingestion and suggest that simply upgrading household WASH facilities will not reduce infection without addressing the burden of contamination from animals, alongside adequate separation in the home.

Keywords

Campylobacter, Ethiopia, infection, malnutrition, poultry, stunting, WASH.

1. Introduction

Infant growth, infection and domestic animal exposure

Enteropathogen infection and associated diarrhoea in infancy and the relationship with linear growth failure (stunting) is a dynamic area of research in infant malnutrition. Whilst child deaths from diarrhoea dropped by over half in just 15 years between 2000−2015[1], diarrhoeal episodes have not similarly decreased[2] suggesting a need for better measures to detect and prevent infection. Early diarrhoea and diarrhoea-related sequelae hold both acute and chronic consequences. Whilst good evidence indicates that a heavy early diarrhoeal burden does affect growth and worsen nutritional status[3–5], there is debate about its relative contribution to long-term growth faltering[6,7]. Other direct, biological causes under study include environmental enteric dysfunction (EED): a condition characterised by the disturbance of gut immunity, structure and function, which ultimately impairs nutrient absorption and linear growth – even without diarrhoea[8–10]. Nonetheless, the common underlying factor to these different contributors is early exposure to pathogenic bacteria and repeated infection[11,12]. As such it is increasingly evident that stunting will not be resolved by improved nutritional intake or acute rehabilitation alone[13] but with parallel improvements in water quality, sanitation and hygiene (WASH) which act as a primary barrier to infection.

Recent cluster-randomised control trials have sought to investigate the effect of improved WASH, alone and in combination with nutrition supplementation, on child health. However different study designs and settings have for the most part failed to show consistent evidence for a reduction in diarrhoea or improvements in malnutrition indicators[14–19]. One

possibility is that despite thorough design, interventions mainly focused on containing human excreta and did not consider (and conventionally have not considered) the role of animal faeces in domestic contamination and illness:[20] surprising given over 60% of infectious diseases in humans are caused by zoonotic pathogens[21]. Transmission pathways are not mutually exclusive, and inadequate separation of animals from the home environment may inevitably result in faecal-oral transmission through direct contact with animal faeces or contaminated soil, or faecal contamination of hands, food, objects or water sources[22–24]. Infants are also vulnerable to transmission routes specific to age-related behaviours, including contaminated floors, where they crawl and directly or indirectly ingest faecal material[25–27]. As such, animal faecal contamination is a neglected factor potentially contributing to infection, diarrhoea and linear growth failure.

Infant *Campylobacter* infection and transmission

Previous studies have sought to understand the disease burden attributed to animal faeces which acts as a transmission vector via the faecal-oral pathway[20–22]. Key zoonotic pathogens related to infant infection, growth failure and EED include G*iardia*[28,29]*, enteroaggregative* and *enteropathogenic E. coli*[28–30], *Shigella*[31,32] and *Cryptosporidium*[33,34] which are transmitted across multiple pathways within the home and ingested through normal infant hand-to-mouth behaviour[25,35]. Among those pathogens of highest concern, *Campylobacter* consistently emerges as one of the key contributors to diarrhoea and malnutrition[31,32,34] and EED[28]. One of the most widespread infectious diseases, Campylobacteriosis is endemic across lower-income countries, especially in children[36] – responsible for 30,931 diarrhoeal-related deaths in 2015[37]. The infectious dose for Campylobacteriosis is low compared to other bacterial infections, with reported minimum values of around 500 CFU leading to infection in adults:[38,39] this value may also be lower for infants where immune systems are immature. Infection is acute and generally self-limiting: however while mean excretion is reported at around seven days[36], the bacteria has been isolated from faeces up to two weeks following infection[40,41]. Prolonged excretion may enhance transmission and incidence[42] and where it

also affects the epithelial barrier[43] may contribute to gut mucosal damage and other EED-like abnormalities[44].

Large studies across many different low-income settings have attributed both asymptomatic and symptomatic *Campylobacter* infection with shorter length attainment of up to one centimetre[32,44] and with changes in EED clinical markers[43,45]. Thermophilic *C. jejuni* (~90%) and *C. coli* are the most commonly isolated *Campylobacter* species in diarrhoeal disease[46], and as part of the normal intestinal flora of birds, poultry represents one of the major sources of transmission, contamination and infection[47]. An essential component of livelihoods and nutrition security, poultry ownership – particularly chickens – is ubiquitous across many low-income nations[48]. Largely free-ranging and dependent on scavenging, chickens frequently openly defecate inside the home and so infants are frequently exposed to, and often consume, chicken faeces and/or contaminated floor surface material during crawling or play[27,49,50]. As domestic floors are usually made of compacted soil, detection and removal of small poultry faeces is difficult and so Campylobacteriosis risk in crawling infants is high. Beyond six months of age critical developmental stages of weaning and crawling mean infection risk increases,[51] with obvious implications for short- and long-term growth and development. However, the evidence base describing the links between domestic animal ownership (particularly chickens), WASH facilities and use and infant nutritional status is limited to a few observational studies[27,52–55], which have not consistently measured *Campylobacter* carriage and/or infection. There is insufficient evidence to fully describe the extent to which infection is caused by exposure to domestic animals in low- and middle-income countries, and furthermore, if infant nutritional status affects whether infection is clinical or sub-clinical.

Study aims

Thus there is a need for further research which describes *Campylobacter* prevalence in young infants and the relationship to animal ownership and health outcomes, whilst also

considering household WASH facilities and use. Further data is also needed on infection and age-related transmission pathways, including domestic floors which are of high risk to this age group[56–58]. This small study aimed to provide formative evidence toward the prevailing hypothesis that infant health is negatively associated with stools positive for *Campylobacter* and exposure to domestic animals, whilst not mitigated by WASH facilities. It aimed to determine: i) Infant *Campylobacter* prevalence in a sample of rural, subsistence households in Sidama zone, Ethiopia with domestic animals ii) The relationship between both asymptomatic and symptomatic *Campylobacter* positive infants and anthropometric indices across households and iii) Risk factors and possible transmission pathways associated with infants positive for *Campylobacter*.

2. Methods

Country context and study sample

In Ethiopia, despite substantial recent reductions, linear growth failure affected more than a third of infants in 2016[59]. Ethiopia has one of the highest domestic animal densities per km^2 worldwide[60] and poultry are ubiquitous in rural households. Some research in Ethiopia has documented the proximity and exposure of infants to chickens and their faeces in regions[61] and the relationship with infant growth[54], and a few regional studies have associated *Campylobacter* infection with infant diarrhoea and malnutrition.[62–64]. However further research is required in Ethiopia on the epidemiology of infant *Campylobacter* prevalence and infant health outcomes and the relationship to poultry ownership and WASH facilities. This small, formative study was conducted in the Southern Nations, Nationalities, and Peoples' region (SNNPR), Sidama zone (regional subdivision), Ethiopia, as the geographical outreach area of the non-governmental organisation People in Need. The study took part in the month of June 2019 – the start of the region's rainy season. Two rural kebeles (neighbourhoods) were chosen from a woreda (zonal subdivision) which remained representative of typical rural livelihoods across Sidama zone. A simple random sampling

method was used to identify households fulfilling the eligibility criteria of having an infant aged 10−18 months and owning free-range poultry. The random sample is described as follows. After communication with a government Health Extension Worker (HEW) local to each kebele, the team produced a sampling frame for both kebeles of all infants aged 10−18 months from households known by the HEW to own poultry. For both sampling frames, households were sequentially numbered on paper and using a simple lottery method 17 and 18 infants were randomly drawn from the two kebele frames respectively for a total sample of 35 infants. Households were visited on a single occasion.

As this study was designed to provide formative evidence, a sample size calculation was not performed. Formative research is often conducted as part of the process of a larger study design and provides data for research teams to plan interventions or further data collection. Formative research is early phase data and is not powered to detect differences between groups. As such, this study results must be interpreted in this context, where it provided indicative data towards the hypothesis but was not sufficiently powered for conclusive evidence[65].

Survey and anthropometry

A survey previously validated in the region[50] assessed latrine type and use, handwashing practices and soap availability, domestic animal ownership and husbandry practices and infant diarrhoeal prevalence and duration. To assess diarrhoea, caregivers were asked the frequency of loose or watery stools during the last day and over the past seven days. World Health Organisation (WHO) criteria was applied retrospectively, where diarrhoea is defined as at least three loose or watery stools within a 24-hour period[66]. Reported diarrhoea was later compared with the quality of stool samples, where all cases of reported diarrhoea matched visible diarrhoeal stool consistency. Presence and evidence of use of a working latrine and handwashing station were also validated by direct observation. After primary introductions with the caregivers and informed consent, a fieldworker completed the survey

with translation from the HEW. Anthropometry measures were infant recumbent length (measured to the nearest 0.1 cm) and weight (measured to the nearest 100 g), taken by trained personnel following standard procedures[67] using a hanging Salter scale and a portable, fixed base length board.

Sample collection and transport

A day prior to household visits, HEWs distributed sterile sample collection bags with a sterile scoop to households for faecal sample collection (Whirl-Pak® WPB01478WA, Sigma-Aldrich, UK). Caregivers were shown how to use the sterile scoop and seal the bags to minimise contamination, and were requested to collect a fresh faecal sample from their poultry and infant as close as possible to sample collection within 24 hours. Households were instructed to collect poultry samples from inside the home. During the study visit a third sample was collected from the floor surface inside the home. The infant's mother was asked to indicate the location the infant usually plays, and a researcher collected a sample of compacted floor surface (approximately 20 g) into another collection bag. All samples were transported in an insulated cool bag on ice to the laboratory at Hawassa University College of Medicine and Health Sciences within five hours. Upon arrival to the laboratory, samples were stored refrigerated (2−8°C) prior to analysis and plates were inoculated and incubated within two hours of arrival to the laboratory. Plates were discarded safely following successful growth. Sample collection and transport methods echo similar methods in studies conducted in Ethiopia[64]. Thus each household sampling event (N=35) comprised three samples (poultry and infant faeces and floor surface). Due to damaged collection bags, three floor surface samples were discarded to give a total of 102 samples analysed for presumptive *Campylobacter* spp. Samples were numbered anonymously which linked the relevant household but removed all identifiers.

Isolation of *Campylobacter* spp.

Presumptive thermotolerant *Campylobacter* spp. was isolated from fresh faecal samples from poultry, infants and floor surface samples. Methods were briefly as follows. Aseptic techniques were followed and samples weighed using sterile disposable weighing boats to 1 ± 0.05 g wet weight. Samples were then aliquoted into sterile plastic centrifuge tubes containing 9 mL of prepared sterile peptone water and vortexed well. For poultry faecal samples only, 100 µL of sample was pipetted into sterile tubes containing 900 µL of peptone water to prepare a 10-fold serial dilution up to 10^5 dilution. 100 µL of floor surface and infant faecal samples and poultry faecal sample dilutions of orders 10^1, 10^3 and 10^5 were drop plated on pre-labelled plates and spread using disposable L-shaped spreaders. Blood-free chromogenic CHROMagar™ Campylobacter media (CHROMagar™, France) was used for the selective detection and differentiation of presumptive thermotolerant *Campylobacter*, prepared and used according to manufacturer instructions[68]. Inoculated plates were allowed to dry under a laminar flow for approximately five minutes as per manufacturer instructions, inverted and stacked into anaerobic jars and incubated at 42°C for 48 hours under microaerophilic conditions. CampyGen™ 2.5 L sachets (Thermo Scientific™, UK) were used to obtain a hydrogen-free microaerophilic atmosphere of approximately 5% O_2, 10% CO_2 and 85% N_2, suitable for the growth of *Campylobacter* spp.

Identification of *Campylobacter* spp.

After 48 hours, presumptive *C. jejuni*, *C. coli* and *C. lari* appear on the chromogenic agar as intense red coloured colonies on a translucent base. Other non-target microorganisms are inhibited (i.e. small, blue colour or absent colonies[68]) and high specificity and sensitivity versus other media is well demonstrated[69–71]. Quality control and preparation of the medium was tested by isolating the ATCC® strain *C. jejuni* (33291) under representative conditions at Cranfield University prior to fieldwork. Blank samples with no growth confirmed no external contamination in all batches.

Ethics

At the start of each household visit, the study was introduced by the field team and HEW and informed consent was described to the caregiver in their first language of Amharic or Sidamo. Fieldworkers tested the caregivers' understanding of consent by asking them questions regarding the study and the consent process, and explained all data was anonymised. As most adult caregivers were illiterate, oral consent and assent for their infant was recorded. The survey was written in English, translated to Amharic by the field team and verbally translated into Sidamo by a HEW. The study protocol was approved by two institutional review boards: Cranfield University Research Ethics Committee (CURES/7774/2019) and Hawassa University College of Medicine and Health Sciences (IRB/222/11).

Statistical analysis

Analyses were performed at the household level. Plates were visually inspected for presumptive *Campylobacter* spp. and recorded as growth or non-growth and the prevalence (as percentage) of positive poultry, infants and floor surfaces was calculated. Whilst the presence of *Campylobacter* does not necessarily indicate active infection, for the purpose of analysis, samples with presumptive *Campylobacter* growth were classified as 'positive' or with no growth as 'negative'. Positive infant faecal samples were then described as symptomatic (the positive stool sample was diarrhoeal), or asymptomatic (the stool sample was not diarrhoeal). Z scores were calculated for length-for-age and weight-for-length (LAZ and WLZ respectively) using the WHO 2006 Child Growth Standards[72]. Z-scores were categorised into stunting and wasting using the standard cut-off value less than −2 standard deviations of the reference[72]. Anonymised household survey data were entered into Microsoft Excel, coded for descriptive analysis and further analysed using SPSS (version 22.0, IBM, New York). Simple frequency distribution tests described survey response data, anthropometric data and *Campylobacter* prevalence. Fisher's exact test for independence tested associations between variables for the small sample size (5% significance). Results

with significant p-values from the Fisher's exact test reported odds ratio (OR) risk estimates with corresponding 95% confidence intervals (CI).

3. Results

Survey and anthropometric data

Data were collected from all 35 households identified in the sampling frame. Results from the survey and anthropometric data are shown in Table 1. Average infant age was 15 months. Almost a third (31.4%) of infants had experienced diarrhoea within the past 7 days with an average duration of 3.1 days. 88.6% of households owned a latrine, most of which were improved pit latrines with a slab (82.9%). Less than half (40.0%) of households had some form of handwashing facility available (including a simple basin and jug) and half (51.4%) owned soap. Aside from poultry ownership, cattle was the second most common form of animal husbandry (n=19, 54.3%). Regarding animal husbandry practices 97.1% of households reported that during the day their animals shared the same living space as the family, and 91.4% during the night. Mean WLZ score was -0.61 (range -2.14−0.64, SE 0.15) and mean LAZ score was -0.81 (range -2.53−0.94, SE 0.19). Overall, five infants (14.3%) were classified as wasted (WLZ <-2 SD), eleven (31.4%) as stunted (LAZ <-2 SD) and four infants both wasted and stunted (11.4%, WLZ and LAZ <-2 SD). Of those infants classified as wasted (n=5), all had experienced diarrhoea within the past seven days ($p<0.001$; OR 1.83, 95% CI 1.07−3.14). Diarrhoeal prevalence was not significantly related to stunting ($p=0.709$).

Table 1. Infant and household characteristics (N=35) including animal husbandry practices and anthropometric indicators, Sidama zone, Ethiopia.

Household characteristic	n	Average or percent (%) of total
Infant sex		
Male	19	54.3%
Female	16	45.7%
Average age (months)		15
Diarrhoea during the last 7 days	11	31.4%
Average duration of diarrhoea (days)		3.7
Household owns a latrine	31	88.6%
Household latrine type		
Open defecation (no latrine)	1	2.9%
Use neighbour's toilet (no latrine)	3	8.6%
Pit latrine without slab	2	5.7%
Pit latrine with slab	29	82.9%
Household has a handwashing facility	14	40.0%
Household has soap available	18	51.4%
Household domestic livestock ownership		
Chickens	35	100%
Cattle	19	54.3%
Goats	11	31.4%
Donkey(s)	2	5.7%
Livestock practices during the day:		
Live outside	35	100.0%
Live inside in the same room as the family	34	97.1%
Live inside in a separate room to the family	1	2.9%
Livestock practices during the night:		
Live inside in the same room as the family	32	91.4%
Live inside in a separate room to the family	3	8.6%
**Nutrition indicator**	**n**	**Percent (%) of total**
Weight-for-length (WLZ)		
-2 to -3 SD (wasted)	5	14.3%
Length-for-age (LAZ)		
-2 to -3 SD (stunted)	11	31.4%
WLZ and LAZ		
-2 to -3 SD (stunted and wasted)	4	11.4%

Campylobacter prevalence and correlation with infant health measures

The following sections describe the relationships between survey variables, prevalence of presumptive *Campylobacter* and infant health outcomes. A total of 102 samples from poultry, infants and floor surface were cultured for *Campylobacter* spp. Overall, *Campylobacter* was recovered from 48.6% (n=17) of 35 infant faecal samples, 68.6% (n=24) of 35 poultry faecal samples and 65.6% (n=21) of 32 floor surface samples. Differences in the prevalence of positive samples which were symptomatic (a diarrhoeal stool sample) and asymptomatic (non-diarrhoeal stool, 'carriers') was seen among positive infants presenting with diarrhoeal stools (n=10, 58.8%) versus without diarrhoea (n=7, 41.2%) ($p<0.001$). Furthermore, infant who were wasted (low weight-for-length) versus not wasted were compared for *Campylobacter* prevalence. Those wasted were more likely to test positive for *Campylobacter* ($p=0.019$; OR 1.41, 95% CI 1.04−1.92). Wasted infants thus appeared to have a 1.83 times the odds of diarrhoea and 1.41 times of a sample positive for *Campylobacter* versus those not wasted. However, diarrhoea was not associated with infant stunting ($p=0.709$), nor was *Campylobacter* prevalence ($p=0.725$).

Risk factors and transmission pathways related to infant _Campylobacter_ prevalence

Further analysis using correlation explored the relationship between potential risk factors and transmission pathways to infant stools positive for *Campylobacter* . Considering associated risk factors, animal husbandry practices of keeping animals inside during the day and night (as a composite variable) was strongly correlated with increased odds of infants positive for *Campylobacter* ($p=0.027$, OR 3.5, 95% CI 1.31–8.77). Owning donkeys or goats showed no association ($p=0.229$ and $p=0.546$ respectively), but owning cattle was significantly associated with increased odds, although with high uncertainty of effect ($p=0.018$, OR 6.5, 95% CI 1.47−28.90). Poultry faeces positive for *Campylobacter* showed significant correlation with infant *Campylobacter* ($p<0.001$, OR 1.34, 95%CI 1.21−1.69). However, owning a latrine, different types of latrine, owning a handwashing facility and ownership of soap were all not correlated (all $p>0.5$). Considering potential transmission

pathways, positive floor samples showed a significant association, although again with high uncertainty of effect (p=0.023, OR 7.0, 95% CI 1.5−23.4). Positive poultry faeces and positive floor samples were also highly correlated (p=0.013; OR 2.68. 95% CI 1.64−12.62). The associations between risk factors and transmission pathways in relation to infant health outcomes are detailed in Table 2. Fig. 1 also illustrates these associations whereby the dotted lines describe the main transmission pathways to an infant stool positive for *Campylobacter*.

Table 2. Odds ratios (OR) for exposure measures predicting an infant stool positive for *Campylobacter*, with corresponding confidence intervals (CI) and p values (significance < 0.05).

**Variable**	**OR**	**95% CI**	**P value**
Infant wasting (WLZ <−2 SD)	1.41	1.04−1.92	0.019
Positive poultry faeces	1.34	1.21−1.69	<0.001
Keeping animals inside (day and night)	3.50	1.31–8.77	0.027
Owning cattle	6.50	1.47−28.90	0.018
Positive floor sample	7.00	1.50−23.40	0.023

Fig 1. Diagram exploring pathways between variables that predict infant stools positive for Campylobacter *and the relationship with health outcomes. Dotted lines demonstrate the hypothesised pathway linking poultry ownership,* Campylobacter prevalence *and health outcomes via clinical or subclinical disease. 'Symptomatic' infection refers to infants positive for Campylobacter who also had a diarrhoeal stool. P values <0.05 were deemed significant. This diagram is expanded in Fig 2. CB, Campylobacter; WLZ, weight-for-length; LAZ, length-for-age.*

4. Discussion

Results from this small cross-sectional study suggest that in these rural Sidamo households raising free-range domestic poultry, the prevalence of infants testing positive for *Campylobacter* spp. is high. With presumptive *Campylobacter* isolated in almost half of infant stools, results mirror high prevalence found in similar age infants in Zimbabwe (32.3%)[73], Mexico (66.0%)[42], Madagascar (43.3%)[74] and across eight low-resource settings where 84.9% of infants had at least one positive faecal sample by one year of age[45]. The high prevalence in this study may be due to sample collection during the rainy season where pooled water inside the home facilitates the spread of faecal bacteria: however other studies have found constant high prevalence not affected by seasonality[44,75]. In this study, 58.8% of the 17 infants positive for *Campylobacter* were symptomatic with diarrhoeal stools. With an average *Campylobacter* excretion of seven days[36] (and reported protracted excretion of more than 14 days[40]) this may lend support that current diarrhoea in these infants was from Campylobacteriosis. Studies in northern Ethiopia[62,63] and in the same zone as this study[64] suggest *Campylobacte*r is a major regional cause of diarrhoea Comparing infants who were wasted (n=5) versus those non-wasted, wasting was correlated with positive *Campylobacter* prevalence and diarrhoeal stools. *.* Campylobacteriosis may have contributed to these outcomes, but it is likely other coexisting infections also contributed[32,76].

Whilst in early infancy infection may produce clinical symptoms and affect short-term weight, repeated enteropathogens colonisation may contribute to the development of EED. Although this study was not able to collect biological measures of EED, 41.2% (n=7) of positive infant stools were asymptomatic (non-diarrhoeal stools). This supports findings from the MAL-ED study where subclinical infection was more strongly related to growth failure than overt diarrhoea[12]. Although positive stools showed no significant correlation with stunting, this may be partly due to the small sample size. Furthermore, research suggests that growth shortfalls resulting from early exposure to *Campylobacter* manifests later in infancy[31]. Studies have

associated cohabiting with poultry with reduced length-for-age[53,77] and others have shown that infants who frequently test positive for *Campylobacter* have lower LAZ scores at 24 months of age, which had a stronger correlation with subclinical infection, or *Campylobacter* carriage[31,44]. Other studies have also demonstrated a relationship between poultry ownership and lower WAZ but not lower LAZ[53], suggesting both acute and chronic effects on health. Other significance lies in the overlap between wasting and stunting among infants in this group (p=0.026), supporting evidence that the two forms of malnutrition can, and often do, coexist in the same infant[78], that they may share common causal factors of repeated carriage and/or infection[79].

This study aimed to further describe the relationship between domestic animal ownership and infant pathogen prevalence and growth, where free-roaming domestic animals may contribute to contamination of the home environment with pathogenic bacteria. Indeed in this study, households were instructed to collect poultry samples from indoors and only two collected samples from outdoors, highlighting the ubiquity of poultry faeces inside the home. Infection is possibly transmitted to infants via age-specific behaviours and pathways. In this study, the significant risk factors that correlated with positive infant stools were specific animal husbandry practices of keeping animals inside during the day and night (ubiquitously in the same room as the family), owning cattle, positive domestic floor samples and positive poultry faeces. The analysis showed some uncertainty of effect and the small sample size may reduce the validity of findings, however the results do highlight specific risk factors to infants, including contaminated domestic floors as a potentially important transmission pathway. Longitudinal data from the MAL-ED team showed the effect of *Campylobacter* infection on growth is related to age – highlighting an increased level of risk as infants start to crawl[31]. Whilst this study did not capture hand-to-mouth contact events, previous research by this team in the same geographical area recorded infants mouthed their own hands or those of their caregiver a mean 31 and 21 times respectively over one hour, which were often visibly dirty (90.0% and 86.0% respectively)[50]. In the same study 35.0% of infants

directly ingested floor surface material and poultry faeces was directly ingested by two infants (10.0%)[50]. Other studies have also recorded infants frequently ingesting poultry faeces from the floor during normal exploratory play[27,80,81].

Other factors not measured in this study, such as contaminated hands, food (particularly milk) and drinking water may account for the remaining sources of and transmission pathways to infant infection. Although a fastidious organism, *Campylobacter* is widespread in the environment, transmitted particularly through contaminated groundwater and stored drinking water[46], surviving for several days in an ambient environment[49]. Consequently research has suggested that in households where poultry are free-roaming, even with good water supply it is unlikely handwashing will effectively interrupt transmission[49]. *Campylobacter* transmission is also increased when WASH facilities are poor:[36] similar cross-sectional studies in Ethiopia also found higher *Campylobacter* prevalence in households without clean water and which had direct contact with chickens[82,83]. In this study latrine ownership and type (improved or not), ownership of handwashing facilities and soap were not correlated with stool samples negative for *Campylobacter*, perhaps suggesting that simply providing WASH facilities will not prevent transmission and infection. However it is possible facilities are also not used, particularly by children, which remains a limitation. In rural communities it can be difficult to assess and accurately report the use of latrines and soap for handwashing. Whilst in this study the visual inspection of latrines suggested they were all used, soap ownership would often be reported but not seen. Regardless, it seems logical that when sharing living spaces so closely, domestic animals contribute to infection from zoonoses and widespread contamination of multiple pathways . There are intrinsic and inseparable connections between these various transmission pathways. This is illustrated in Fig 2, which illustrates causal pathways to poor infant health outcomes when animal faecal contamination and age-specific infant behaviours are not considered as important risk factors.

Fig 2. The hypothesised pathways by which domestic poultry ownership contributes to acute and chronic infant malnutrition via infection from, and transmission of, Campylobacter. The thicker part of the diagram illustrates the hypothesised relationship with environmental enteric dysfunction (EED). The dotted part of the diagram to the upper right constitutes the original 'F diagram', representing other transmission pathways by which infants are exposed to Campylobacter. Adapted alongside a previously published diagram[9] *and the 'F published by Wagner, E. and Lanoix, J., 1958*[84]*.*

The validity and broader applicability of findings from this study are mostly limited by the small sample size which may affect data validity and generalisability of the results. The single time point of testing in this formative research and the cross-sectional study design prevent determining causality. However, the results are emphasised as formative evidence, and support emerging hypotheses which associate free-range poultry ownership, household contamination and infant infection with undernutrition. Although this study intentionally sampled households who owned poultry, the risk of transmission may actually be greater than estimated as free-range chickens from neighbouring households may also increase contamination. Also, faecal samples from other domestic animals which also harbour *Campylobacter*, such as cattle[46], were not sampled.. On the other hand there was no evaluation of the prevalence of other pathogenic or parasitic organisms, so it is not certain that the presumptive *Campylobacter* isolated in samples was the definite cause of wasting and/or diarrhoeal prevalence seen here. A few studies have reported mixed infections of *Campylobacter* and viral pathogens and their associations with infant morbidities[32,85]. This presence of *Campylobacter* alongside the carriage of multiple pathogens may correlate as a proxy for infants with greater overall levels of exposure to enteric pathogens in their environment; this in turn may associate with those with poor growth and/or wasting.

Lastly, the use of culture-based method alone holds limitations: firstly due to changes in *Campylobacter* cell physiology and loss of viability between sample deposition, collection,

transport and plating (whereby cells enter the viable but non-cultivable [VBNC] state). This may have underestimated the true prevalence. On the other hand, culture holds limited sensitivity and high rates of false detection;[86] whilst there is evidence for good specificity of the agar in comparison and evaluation studies, there is no certainty of the rate of false positives in this study. Lastly, whilst the culture media shows high specificity, it was not possible to differentiate between or quantify different *Campylobacter* species. The parallel use of qPCR alone or PCR with ELISA methods would enhance culture-based findings[31,87].

Conclusion

This formative study adds further preliminary evidence to the body of research documenting infant *Campylobacter* carriage and infection in households rearing free-range poultry. In these households, increased wasting and diarrhoea was seen in infants positive for presumptive *Campylobacter*. Repeated symptomatic infection and low weight may mean infants risk entering a spiral of weight loss and subsequent growth deficits. Alternatively, frequent carriage, or asymptomatic infection, and a high prevalence of stunting (although not correlated) suggest a longer-term impact of exposure to *Campylobacter* that may operate through EED. The time frame for when, and thresholds at which repeated *Campylobacter* infection becomes subclinical, contributes to the development of EED and affects growth are important remaining questions which a larger prospective cohort might address.

More broadly, this study also contributes to discussions around general WASH facilities and use, living conditions and the impact on reducing pathogen transmission. Where contaminated domestic floors are a risk factor for pathogen transmission to infants[56,88] and WASH facilities also appear have little effect in mitigating transmission, this emphasises the high thresholds of hygiene and living conditions necessary to improve infant health. While improvements to basic WASH usually included in interventions may address some secondary pathogen transmission routes, a remaining burden of infection may be expected when animals share the living space. An extensive, multifaceted approach to improve infant

health will require not only improved WASH facilities, but working with communities to adapt current animal husbandry practices, encourage the safe handling and disposal of both animal and adult/infant faeces, safe preparation and storage of food, handwashing with soap after animal/faecal contact and education on the health risks of infant exposure. These multiple, concurrent needs form the rationale for the recent push toward 'transformative WASH'[89] or 'WASH++'[90]. Future research in the WASH sector must develop and test transformative WASH interventions if we are to achieve the high hygiene thresholds that support optimal infant growth.

Acknowledgements

Sophie Budge wrote the manuscript. All other authors assisted in assessing both the paper quality and contributed to the writing and review of the manuscript. The authors wish to thank all of the People in Need team at the Hawassa office who assisted in data collection, logistics and planning. They would also like to thank Sara and Abebe at Hawassa University College of Medicine and Health Sciences and the wider faculty staff who gave their space and time. Finally the authors thank all of the study participants who kindly welcomed us into their homes and offered their time.

Declaration of interest

All authors have no conflict of interest to declare.

Data access

All data created during this research is openly available from the Cranfield Online Research Data at 10.17862/cranfield.rd.9907385.

Supporting information legends

S1 Checklist: STROBE Checklist

Survey 1.

References

1. Liu L, Oza S, Hogan D, et al. Global, regional, and national causes of under-5 mortality in 2000–15: an updated systematic analysis with implications for the Sustainable Development Goals. *Lancet*. 2016;388(10063):3027-3035. doi:10.1016/S0140-6736(16)31593-8
2. Fischer Walker CL, Perin J, Aryee MJ, Boschi-Pinto C, Black RE. Diarrhea incidence in low- and middle-income countries in 1990 and 2010: A systematic review. *BMC Public Health*. 2012;6(3):E230-E231. doi:10.1186/1471-2458-12-220
3. Danaei G, Andrews K, Sudfeld C et al. Risk Factors for Childhood Stunting in 137 Developing Countries: A Comparative Risk Assessment Analysis at Global, Regional, and Country Levels. *PLoS Med*. 2016;13(11):1–18.
4. Troeger C, Colombara D V., Rao PC, et al. Global disability-adjusted life-year estimates of long-term health burden and undernutrition attributable to diarrhoeal diseases in children younger than 5 years. *Lancet Glob Heal*. 2018;6(3):e255-e269. doi:10.1016/S2214-109X(18)30045-7
5. Guerrant RL, Oriá RB, Moore SR, Oriá MOB, Lima AAAM. Malnutrition as an enteric infectious disease with long-term effects on child development. *Nutr Rev*. 2008;66(9):487-505. doi:10.1111/j.1753-4887.2008.00082.x
6. Bhutta Z, Ahmed T, Black R et al. What works? Interventions for maternal and child undernutrition and survival. *Lancet*. 2008;371(9610):417–440.
7. Lunn P, Northrop-Clewes C, Downes R. Intestinal permeability, mucosal injury, and growth faltering in Gambian infants. *Lancet*. 1991;338(8772):907–910.
8. Keusch GT, Denno DM, Black RE, et al. Environmental enteric dysfunction: Pathogenesis, diagnosis, and clinical consequences. *Clin Infect Dis*. 2014;59(S4):S207–S212. doi:10.1093/cid/ciu485
9. Budge S, Parker AH, Hutchings PT, Garbutt C. Environmental enteric dysfunction and child stunting. *Nutr Rev*. 2019;77(4):240–253. doi:10.1093/nutrit/nuy068

10. Owino V, Ahmed T, Freemark M et al. Environmental Enteric Dysfunction and Growth Failure/Stunting in Global Child Health. *Pediatrics*. 2016;138(6):e20160641–e20160641.
11. Ahmed T, Auble D, Berkley J et al. An evolving perspective about the origins of childhood undernutrition and nutritional interventions that includes the gut microbiome. *Ann N Y Acad Sci*. 2014;1332(1):22–38. doi:10.1111/nyas.12487
12. MAL-ED Network Investigators. Relationship between growth and illness, enteropathogens and dietary intakes in the first 2 years of life: Findings from the MAL-ED birth cohort study. *BMJ Glob Heal*. 2017;2(4):e000370. doi:10.1136/bmjgh-2017-000370
13. Dewey K, Adu-Afarwuah S. Systematic review of the efficacy and effectiveness of complementary feeding interventions in developing countries. *Mater Child Nutr*. 2008;4(S1):24–85. doi:10.1111/j.1740-8709.2007.00124.x
14. Luby SP, Rahman M, Arnold BF, et al. Effects of water quality, sanitation, handwashing, and nutritional interventions on diarrhoea and child growth in rural Bangladesh: a cluster randomised controlled trial. *Lancet Glob Heal*. 2018;6(3):e302-e315. doi:10.1016/S2214-109X(17)30490-4
15. Null C, Stewart CP, Pickering AJ, et al. Effects of water quality, sanitation, handwashing, and nutritional interventions on diarrhoea and child growth in rural Kenya: A cluster-randomised controlled trial. *Lancet Glob Heal*. 2018;6(3):e316–e329. doi:https://doi.org/10.1016/S2214-109X(18)30005-6
16. Humphrey JH, Mbuya MNN, Ntozini R, et al. Independent and combined effects of improved water, sanitation, and hygiene, and improved complementary feeding, on child stunting and anaemia in rural Zimbabwe: a cluster-randomised trial. *Lancet Glob Heal*. 2019;7(1):e132–e147. doi:10.1016/S2214-109X(18)30374-7
17. Clasen T, Boisson S, Routray P, et al. Effectiveness of a rural sanitation programme on diarrhoea, soil-transmitted helminth infection, and child malnutrition in Odisha, India: A cluster-randomised trial. *Lancet Glob Heal*. 2014;2(11):e645-e653.

doi:10.1016/S2214-109X(14)70307-9

18. Cameron L, Olivia S, Shah M. Scaling up sanitation: Evidence from an RCT in Indonesia. *J Dev Econ*. 2019;138:1-16. doi:10.1016/j.jdeveco.2018.12.001

19. Patil SR, Arnold BF, Salvatore AL, et al. The effect of India's total sanitation campaign on defecation behaviors and child health in rural Madhya Pradesh: A cluster randomized controlled trial. *PLoS Med*. 2015;11(8):e1001709. doi:10.1371/journal.pmed.1001709

20. Delahoy MJ, Wodnik B, McAliley L, et al. Pathogens transmitted in animal feces in low- and middle-income countries. *Int J Hyg Environ Health*. 2018;221(4):661-676. doi:10.1016/j.ijheh.2018.03.005

21. Taylor LH, Latham SM, Woolhouse MEJ. Risk factors for human disease emergence. *Philos Trans R Soc B Biol Sci*. 2001;35(1411):983–989. doi:10.1098/rstb.2001.0888

22. Penakalapati G, Swarthout J, Delahoy M et al. Exposure to Animal Feces and Human Health: A Systematic Review and Proposed Research Priorities. *Envir Sci Technol*. 2017;51(20):11537–11552. doi:10.1021/acs.est.7b02811

23. Baker KK, Senesac R, Sewell D, Sen Gupta A, Cumming O, Mumma J. Fecal Fingerprints of Enteric Pathogen Contamination in Public Environments of Kisumu, Kenya, Associated with Human Sanitation Conditions and Domestic Animals. *Environ Sci Technol*. 2018;52(18):10263-10274. doi:10.1021/acs.est.8b01528

24. Ercumen A, Pickering AJ, Kwong LH, et al. Animal Feces Contribute to Domestic Fecal Contamination: Evidence from E. coli Measured in Water, Hands, Food, Flies, and Soil in Bangladesh. *Environ Sci Technol*. 2017;51(15):8725-8734. doi:10.1021/acs.est.7b01710

25. Morita T, Perin J, Oldja L et al. Mouthing of Soil Contaminated Objects is Associated with Environmental Enteropathy in Young Children. *Trop Med Int Heal*. 2017;22(6):670–678. doi:10.1111/tmi.12869

26. Mattioli MCM, Davis J, Boehm AB. Hand-to-mouth contacts result in greater ingestion of feces than dietary water consumption in Tanzania: A quantitative fecal exposure

assessment model. *Environ Sci Technol*. 2015;49(3):1912-1920. doi:10.1021/es505555f

27. Ngure F, Humphrey J, Mbuya M et al. Formative research on hygiene behaviors and geophagy among infants and young children and implications of exposure to fecal bacteria. *Am J Trop Med Hyg*. 2013;89(4):709–716. doi:10.4269/ajtmh.12-0568

28. George C, Burrowes V, Perin J et al. Enteric Infections in Young Children are Associated with Environmental Enteropathy and Impaired Growth. *Trop Med Int Heal*. 2017;12(10):3218–3221.

29. Kosek M et al. Causal Pathways from Enteropathogens to Environmental Enteropathy: Findings from the MAL-ED Birth Cohort Study. *EBio Med*. 2017;18:109–117. doi:10.1016/j.ebiom.2017.02.024

30. Lima AAM, Soares AM, Filho JQS, et al. Enteroaggregative Escherichia coli Subclinical Infection and Coinfections and Impaired Child Growth in the MAL-ED Cohort Study. *J Pediatr Gastroenterol Nutr*. 2018;66(2):325-333. doi:10.1097/MPG.0000000000001717

31. Rogawski ET, Liu J, Platts-Mills JA, et al. Use of quantitative molecular diagnostic methods to investigate the effect of enteropathogen infections on linear growth in children in low-resource settings: longitudinal analysis of results from the MAL-ED cohort study. *Lancet Glob Heal*. 2018;6(12):e1319-e1328. doi:10.1016/S2214-109X(18)30351-6

32. Platts-Mills JA, Taniuchi M, Uddin MJ, et al. Association between enteropathogens and malnutrition in children aged 6-23 mo in Bangladesh: A case-control study. *Am J Clin Nutr*. 2017;105(5):1132–1138. doi:10.3945/ajcn.116.138800

33. Checkley W, Epstein LD, Gilman RH, Black RE, Cabrera L, Sterling CR. Effects of Cryptosporidium parvum infection in Peruvian children: Growth faltering and subsequent catch-up growth. *Am J Epidemiol*. 1998;148(5):497-506. doi:10.1093/oxfordjournals.aje.a009675

34. Kotloff K, Nataro J, Blackwelder W et al. Burden and aetiology of diarrhoeal disease in

infants and young children in developing countries (the Global Enteric Multicenter Study, GEMS): a prospective, case-control study. *Lancet*. 2013;382(9888):209–222. doi:10.1016/S0140-6736(13)60844-2

35. Perin J, Thomas A, Oldja L et al. Geophagy Is Associated with Growth Faltering in Children in Rural Bangladesh. *J Pediatr*. 2016;178:34–39.

36. Coker AO, Isokpehi RD, Thomas BN, Amisu KO, Larry Obi C. Human campylobacteriosis in developing countries. *Emerg Infect Dis*. 2002;8(3):237-243. doi:10.3201/eid0803.010233

37. Troeger C, Forouzanfar M, Rao PC, et al. Estimates of global, regional, and national morbidity, mortality, and aetiologies of diarrhoeal diseases: a systematic analysis for the Global Burden of Disease Study 2015. *Lancet Infect Dis*. 2017;17(9):909-948. doi:10.1016/S1473-3099(17)30276-1

38. Hara-Kudo Y, Takatori K. Contamination level and ingestion dose of foodborne pathogens associated with infections. *Epidemiol Infect*. 2011;139(10):1505-1510. doi:10.1017/S095026881000292X

39. Black RE, Levine MM, Clements M Lou, Hughes TP, Blaser MJ, Black RE. Experimental campylobacter jejuni infection in humans. *J Infect Dis*. 1988;157(3):472-479. doi:10.1093/infdis/157.3.472

40. Rao MR, Naficy AB, Savarino SJ, et al. Pathogenicity and convalescent excretion of Campylobacter in rural Egyptian children. *Am J Epidemiol*. 2001;154(2):166-173. doi:10.1093/aje/154.2.166

41. Mølbak K, Højlyng N, Gaarslev K. High prevalence of campylobacter excretors among Liberian children related to environmental conditions. *Epidemiol Infect*. 1988;100(2):227–237. doi:10.1017/S0950268800067364

42. Calva JJ, Lopez-Vidal AB, Ruiz-Palacios GM, Ramos A, Bojalil R. Cohort study of intestinal infection with campylobacter in Mexican children. *Lancet*. 1988;1(8584):503-506. doi:10.1016/S0140-6736(88)91297-4

43. Chen ML, Ge Z, Fox JG, Schauer DB. Disruption of tight junctions and induction of

proinflammatory cytokine responses in colonic epithelial cells by Campylobacter jejuni. *Infect Immun*. 2006;74(12):6581-6589. doi:10.1128/IAI.00958-06

44. Lee G, Pan W, Penataro Yori P, et al. Symptomatic and Asymptomatic Campylobacter Infections Associated with Reduced Growth in Peruvian Children. *PLoS Neglected Trop Dis*. 2013;7(1):e2036. doi:10.1371/journal.pntd.0002036

45. Amour C, Gratz J, Mduma ER, et al. Epidemiology and Impact of Campylobacter Infection in Children in 8 Low-Resource Settings: Results from the MAL-ED Study. *Clin Infect Dis*. 2016;63(9):1171-1179. doi:10.1093/cid/ciw542

46. Kaakoush NO, Castaño-Rodríguez N, Mitchell HM, Man SM. Global epidemiology of campylobacter infection. *Clin Microbiol Rev*. 2015;28(3):687-720. doi:10.1128/CMR.00006-15

47. Janssen R, Krogfelt KA, Cawthraw SA, Van Pelt W, Wagenaar JA, Owen RJ. Host-pathogen interactions in Campylobacter infections: The host perspective. *Clin Microbiol Rev*. 2008;21(3):505-518. doi:10.1128/CMR.00055-07

48. FAO. *Decision Tools for Family Poultry Development. FAO Animal Production and Health Guidelines No. 16*. Rome, Italy; 2014.

49. Marquis G, Ventura G, Gilman R et al. Fecal contamination of shanty town toddlers in households with non-corralled poultry, Lima, Peru. *Am J Public Heal*. 1990;80(2):146–149. doi:10.2105/AJPH.80.2.146

50. Budge S, Hutchings P, Parker A, et al. Do domestic animals contribute to bacterial contamination of infant transmission pathways? Formative evidence from rural Ethiopia. *J Water Health*. 2019;17(5). doi:10.2166/wh.2019.224

51. Samuel SO, Aboderin AO, Akanbi AA, Adegboro B, Smith SI, Coker AO. Campylobacter enteritis in Ilorin, Nigeria. *East Afr Med J*. 2006;83(9):478-484.

52. George C, Oldja L, Biswas S et al. Fecal markers of environmental enteropathy are associated with animal exposure and caregiver hygiene in Bangladesh. *Am J Trop Med Hyg*. 2015;93(2):269-275. doi:10.4269/ajtmh.14-0694

53. Gelli A, Headey D, Becquey E, et al. Poultry husbandry, water, sanitation, and

hygiene practices, and child anthropometry in rural Burkina Faso. *Matern Child Nutr*. 2019;e12818. doi:10.1111/mcn.12818

54. Headey D, Hirvonen K. Is exposure to poultry harmful to child nutrition? An observational analysis for rural Ethiopia. *PLoS One*. 2016;11(8):1–16. doi:https://doi.org/10.1371/journal.pone.0160590

55. Ngure F, Gelli A, Becquey E, et al. Exposure to livestock feces and water quality, sanitation, and hygiene (wash) conditions among caregivers and young children: Formative research in rural Burkina Faso. *Am J Trop Med Hyg*. 2019;100(4):998-1004. doi:10.4269/ajtmh.18-0333

56. Exum NG, Olórtegui MP, Yori PP, et al. Floors and Toilets: Association of Floors and Sanitation Practices with Fecal Contamination in Peruvian Amazon Peri-Urban Households. *Environ Sci Technol*. 2016;50:7373−7381. doi:10.1021/acs.est.6b01283

57. George C, Oldja L, Biswas S et al. Geophagy is associated with environmental enteropathy and stunting in children in rural Bangladesh. *Am J Trop Med Hyg*. 2015;92(6):1117–1124. doi:10.4269/ajtmh.14-0672

58. Pickering A, Julian T, Marks S et al. Fecal contamination and diarrheal pathogens on surfaces and in soils among Tanzanian households with and without improved sanitation. *Envir Sci Technol*. 2012;46(11):5736–5743. doi:10.1021/es300022c

59. Central Statistical Agency-CSA/Ethiopia I. *Ethiopia Demographic and Health Survey (DHS) 2016*. Addis Ababa, Ethiopia; 2017. doi:10.2147/ahmt.s148434

60. Sebastian K. *Atlas of African Agriculture Research & Development: Revealing Agriculture's Place in Africa*. Washington DC: International Food Policy Research Institute (IFPRI); 2014. doi:http://dx.doi.org/10.2499/9780896298460

61. Save the Children, The Manoff Group Inc. *Water, Hygiene and Sanitation (WASH) in Rural Households in Amhara, Oromia, SNNP and Tigray. A Report on the Findings and Recommendations of a Multi-Method Qualitative Research Study for Social and Behavior Change Communication Programming in Ethiopia.* Addis Ababa, Ethiopia; 2014.

62. Gedlu E, Aseffa A. Campylobacter enteritis among children in north-west Ethiopia: A 1-year prospective study. *Ann Trop Paediatr*. 1996;16(3):207-212. doi:10.1080/02724936.1996.11747828

63. Mitike G, Kassu A, Genetu A, Nigussie D. Campylobacter enteritis among children in Dembia District, Northwest Ethiopia. *East Afr Med J*. 2009;77(12):654-657. doi:10.4314/eamj.v77i12.46764

64. Mulatu G, Beyene G, Zeynudin A. Prevalence of Shigella, Salmonella and Campylobacter species and their susceptibility patters among under five children with diarrhea in Hawassa town, south Ethiopia. *Ethiop J Health Sci*. 2014;24(2):101-108.

65. Gittelsohn J, Steckler A, Johnson CC, et al. Formative research in school and community-based health programs and studies: "State of the art" and the TAAG approach. *Heal Educ Behav*. 2006. doi:10.1177/1090198105282412

66. Baqui AH, Black RE, Yunus M, Hoque ARA, Chowdhury HR, Sack RB. Methodological issues in diarrhoeal diseases epidemiology: Definition of diarrhoeal episodes. *Int J Epidemiol*. 1991. doi:10.1093/ije/20.4.1057

67. de Onis M, Onyango AW, Van den Broeck J, Chumlea WC, Martorell R. Measurement and standardization protocols for anthropometry used in the construction of a new international growth reference. *Food Nutr Bull*. 2004;25(1):S27-S36.

68. CHROMagar. CHROMagarTM Campylobacter: Instructions for use. Version 3.0.

69. Bensersa-Nedjar D, Zerouki A, Aggoune N, Yamouni F, Henniche F-Z, Chabani A. *Evaluation Comparative de Trois Milieux de Culture Sélectifs ; CHROMagar Campylobacter (CHROMagar), Karmali (Oxoid) et Campylosel (BioMérieux), Pour La Recherche Des Campylobacter Thermotolérants à Partir Des Échantillons Fécaux*. Alger: Algérie; 2017.

70. Forsberg L, Barth C, Malejczyk K, Minion J. *Evaluation of CHROMagar Campylobacter (CAC)*. Regina, SK. S4P 0W5; 2017.

71. De Zutter L, Van Damme I, Mugg P, Ukaegbu K. *Comparison of Four Different*

Selective Media for the Quantification of Campylobacter in Poultry Meat and Rapid Confirmation of Suspect Colonies. Ghent; 2017.

72. WHO Multicentre Growth Reference Study Group. *WHO Child Growth Standards. Length/Height-for-Age, Weight-for-Age, Weight-for-Length, Weight-for-Height and Body Mass Index-for-Age: Methods and Development. Department of Nutrition Health and Development.* Geneva: World Health Organization; 2006.

73. Rogawski McQuade ET, Platts-Mills JA, Gratz J, et al. Impact of Water Quality, Sanitation, Handwashing, and Nutritional Interventions on Enteric Infections in Rural Zimbabwe: The Sanitation Hygiene Infant Nutrition Efficacy (SHINE) Trial. *J Infect Dis*. 2019;jiz179. doi:10.1093/infdis/jiz179

74. Randremanana R V., Randrianirina F, Sabatier P, et al. Campylobacter infection in a cohort of rural children in Moramanga, Madagascar. *BMC Infect Dis*. 2014;14(372):1-9. doi:10.1186/1471-2334-14-372

75. Mason J, Iturriza-Gomara M, O'Brien SJ, et al. Campylobacter Infection in Children in Malawi Is Common and Is Frequently Associated with Enteric Virus Co-Infections. *PLoS One*. 2013;8(3):e59663. doi:10.1371/journal.pone.0059663

76. MAL-ED Network Investigators. Relationship between growth and illness, entereopathogens and dietary intakes in the first 2 years of life. *BMJ Glob Heal*. 2017;2(e000370). doi:10.1136/ bmjgh-2017-000370

77. Headey D, Nguyen P, Kim S, Rawat R, Ruel M, Menon P. Is exposure to animal feces harmful to child nutrition and health outcomes? A multicountry observational analysis. *Am J Trop Med Hyg*. 2017;96(4):961–969.

78. Schoenbuchner SM, Dolan C, Mwangome M, et al. The relationship between wasting and stunting: a retrospective cohort analysis of longitudinal data in Gambian children from 1976 to 2016. *Orig Res Commun Am J Clin Nutr*. 2019;109:1-10. doi:10.1093/ajcn/nqy326.

79. Martorell R, Young MF. Patterns of Stunting and Wasting: Potential Explanatory Factors. *Adv Nutr*. 2012;3(2):227-233. doi:10.3945/an.111.001107

80. Reid B, Orgle J, Roy K, Pongolani C, Chileshe M, Stoltzfus R. Characterizing potential risks of fecal–oral microbial transmission for infants and young children in Rural Zambia. *Am J Trop Med Hyg*. 2018. doi:10.4269/ajtmh.17-0124

81. Yeager B, Lanata C, Lazo F, Verastegui H, Black R. Transmission factors and socioeconomic status as determinants of diarrhoeal incidence in Lima, Peru. *J Diarrhoeal Dis Res*. 1991;9(3):186–193. doi:https://www.jstor.org/stable/23498080

82. Lengerh A, Moges F, Unakal C, Anagaw B. Prevalence, associated risk factors and antimicrobial susceptibility pattern of Campylobacter species among under five diarrheic children at Gondar University Hospital, Northwest Ethiopia. *BMC Pediatr*. 2013;13(82):1-9. doi:10.1186/1471-2431-13-82

83. Tafa B, Sewunet T, Tassew H, Asrat D. Isolation and Antimicrobial Susceptibility Patterns of Campylobacter Species among Diarrheic Children at Jimma, Ethiopia. *Int J Bacteriol*. 2014;2014(560617). doi:10.1155/2014/560617

84. Wagner E, Lanoix J. *Excreta Disposal for Rural Areas and Small Communities.* Vol 39.; 1958.

85. Schnee AE, Haque R, Taniuchi M, et al. Identification of Etiology-Specific Diarrhea Associated with Linear Growth Faltering in Bangladeshi Infants. *Am J Epidemiol*. 2018;187(10):2210–2218. doi:10.1093/aje/kwy106

86. Buss JE, Cresse M, Doyle S, Buchan BW, Craft DW, Young S. Campylobacter culture fails to correctly detect Campylobacter in 30% of positive patient stool specimens compared to non-cultural methods. *Eur J Clin Microbiol Infect Dis*. 2019;38(6):1087–1093. doi:10.1007/s10096-019-03499-x

87. do Nascimento Veras H, da Silva Quetz J, Lima IFN, et al. Combination of different methods for detection of Campylobacter spp. in young children with moderate to severe diarrhea. *J Microbiol Methods*. 2016;128:7-9. doi:10.1016/j.mimet.2016.06.026

88. Lin A, Arnold B, Afreen S et al. Household environmental conditions are associated with enteropathy and impaired growth in rural bangladesh. *Am J Trop Med Hyg*. 2013;89(1):130–137. doi:10.4269/ajtmh.12-0629

89. Pickering A, Null C, Winch P, Mangwadu G, Arnold B, Prendergast A. The WASH Benefits and SHINE trials: interpretation of WASH intervention effects on linear growth and diarrhoea. 2019;7(8):E1139-E1146. doi:10.1016/S2214-109X(19)30268-2

90. Husseini M, Darboe MK, Moore SE, Nabwera HM, Prentice AM. Thresholds of socio-economic and environmental conditions necessary to escape from childhood malnutrition: A natural experiment in rural Gambia. *BMC Med*. 2018;16(1):199. doi:10.1186/s12916-018-1179-3

Figure 1 Click here to access/download;Figure;Fig 1 Budge S 2019.tif

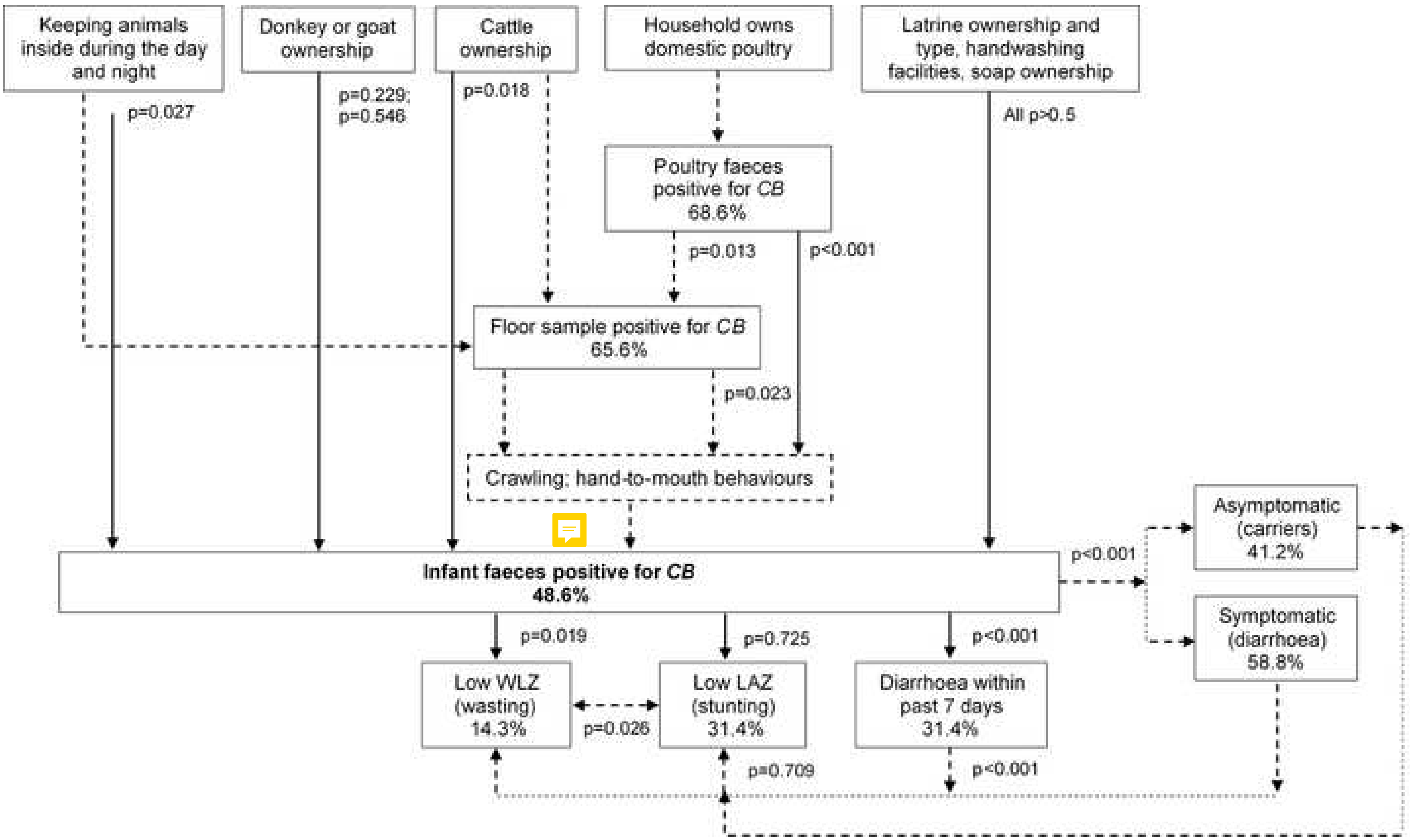

Figure 2 Click here to access/download;Figure;Fig 2 Budge S 2019.tif

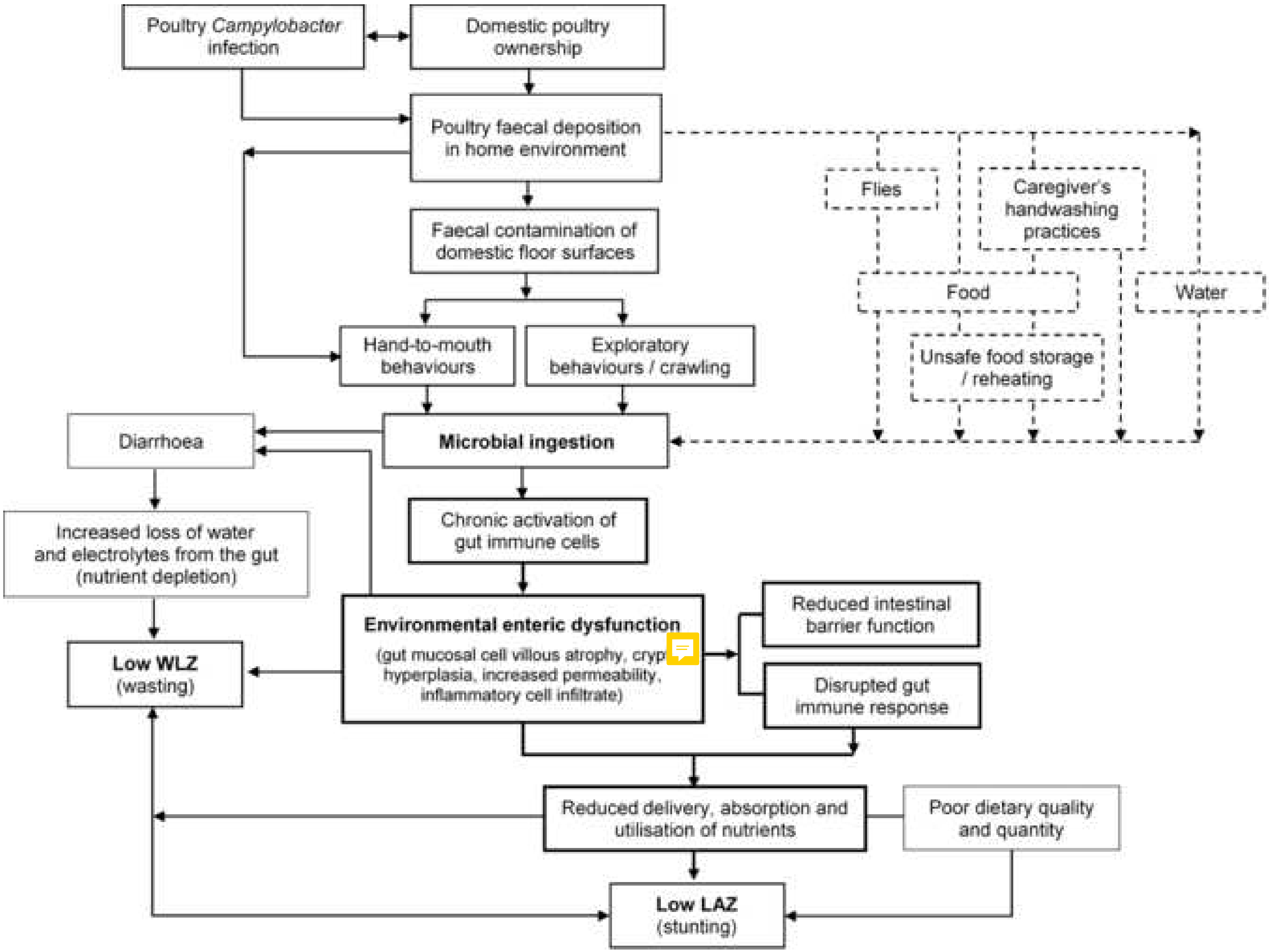

Click here to access/download
Supporting Information
STROBE_checklist_cross-sectional_SBudge_101019.doc

~~Domestic poultry ownership is~~ Risk factors and transmission pathways associated with infant *Campylobacter spp.* ~~infection~~ prevalence and malnutrition: A formative study in rural Ethiopia ~~Formative evidence from Ethiopia~~

Short title

Infant *Campylobacter* ~~infection~~prevalence, malnutrition and domestic animal ownership in rural Ethiopia

Sophie Budge[a¶], Megan Barnett[b¶], Paul Hutchings,[a¶] Alison Parker[a*¶],, Sean Tyrrel[a&], Francis Hassard[a&], Camila Garbutt[c&], Mathewos Moges[d&] Fitsume Woldemedhin[e], Mohammedyasin Jemal[e]

[a] *Cranfield University, College Road, Cranfield, UK.*

[b] *British Geological Survey, Environmental Science Centre, Keyworth, Nottingham, UK.*

[c] *People in Need UK, 49-51 East Road, London, UK.*

[d] *Hawassa University College of Medicine and Health Sciences, Department of Environmental Health, Hawassa, Ethiopia*

[e] *People In Need, Hawassa office, Ethiopia.*

***** Corresponding author

Email: a.parker@cranfield.ac.uk (AP).

¶ These authors contributed equally to this work.

& These authors also contributed equally to this work.

Abstract

Early infection from enteropathogens is recognised as both a cause and effect of infant malnutrition. Specifically, evidence demonstrates associations between growth shortfalls and *Campylobacter* infection, endemic across low-income settings, with poultry a major source. Whilst improvements in water, sanitation and hygiene (WASH) should reduce pathogen transmission, interventions show inconsistent effects on infant health. This cross-sectional, formative study aimed to understand relationships between infant *Campylobacter* prevalence, malnutrition and associated risk factors, including domestic animal husbandry practices, in rural Ethiopia. Thirty-five ~~35~~ households were visited in ~~SNNPR~~ Sidama zone, Southern Nations, Nationalities and Peoples' region. Infant and poultry faeces and domestic floor surfaces (N=102) were analysed for presumptive *Campylobacter* spp. using selective culture. Infant anthropometry and diarrhoeal prevalence, WASH facilities and animal husbandry data were collected. 14.3% of infants were wasted, 31.4% stunted and 31.4% had recent diarrhoea. Presumptive *Campylobacter* spp. was isolated from 48.6% of infant, 68.6% of poultry and 65.6% of floor surface samples. Compared to non-wasted infants, wasted infants had an increased odds ratio (OR) of 1.41 for ~~infection~~ a *Campylobacter*-positive stool and 1.81 for diarrhoea. ~~Infection~~ Positive infant stools showed a significant relationship with wasting (p=0.026) but not stunting. Significant risk factors for ~~infection~~ a positive stool included keeping animals inside (p=0.027, OR 3.5), owning cattle (p=0.018, OR 6.5) and positive poultry faeces (p<0.001, OR 1.34). Positive floor samples showed a significant correlation with ~~infection~~ positive infant (p=0.023), and ~~also correlated with~~ positive poultry (p=0.013, OR 2.68) stools. Ownership of improved WASH facilities was not correlated with lower odds of positive stools. ~~showed no association with reduced infection.~~ This formative study ~~further demonstrates~~ shows a high prevalence of infants positive for *Campylobacter* ~~infection~~ in households with free-range animals. Findings reaffirm contaminated floors as an important pathway to infant pathogen ingestion and suggest that

simply upgrading household WASH facilities will not reduce infection without addressing the burden of contamination from animals, alongside adequate separation in the home.

Keywords

Campylobacter, Ethiopia, infection, malnutrition, poultry, stunting, WASH.

Author summary

Campylobacter is a pathogenic bacteria which is one of four main causes of diarrhoea worldwide. *Campylobacter* is found in food animals like chickens and cattle and transmitted to humans mainly via contaminated food and water. In low-income countries, *Campylobacter* is a common cause of infant diarrhoea and is linked to malnutrition – both wasting (low weight-for-height) and stunting (low height-for-age). However despite evidence for this, we know little about how, why and when infants become infected. This small study tested the theory that infant health is negatively affected by *Campylobacter* infection due to domestic animal exposure, and to assess risk factors associated with infection. We found that in households with free-roaming poultry, almost half of infants had *Campylobacter* infection. 14.3% of infants were wasted, 31.3% were stunted and 31.4% had experienced recent diarrhoea. Risk factors for infection included cattle and poultry ownership and keeping animals inside. Contaminated floor samples were significantly related, highlighting a important transmission pathway to infants who ingest dirty floor material and faeces when crawling. We suggest that without separating animals from living spaces, interventions will not reduce faecal contamination or improve infant growth – even with improvements in water, sanitation and hygiene which should block pathogen transmission.

1. Introduction

Infant growth, infection and domestic animal exposure

Enteropathogen infection and associated diarrhoea in infancy and the relationship with linear growth failure (stunting) is a dynamic area of research in infant malnutrition. Whilst child deaths from diarrhoea dropped by over half in just 15 years between 2000−2015[1], diarrhoeal episodes have not similarly decreased[2] suggesting a need for better measures to detect and prevent infection. Early diarrhoea and diarrhoea-related sequelae hold both acute and chronic consequences. Whilst good evidence indicates that a heavy early diarrhoeal burden does affect growth and worsen nutritional status[3–5], there is debate about its relative contribution to long-term growth faltering[6,7]. Other direct, biological causes under study include environmental enteric dysfunction (EED): a condition characterised by the disturbance of gut immunity, structure and function, which ultimately impairs nutrient absorption and linear growth – even without diarrhoea[8–10]. Nonetheless, the common underlying factor to these different contributors is early exposure to pathogenic bacteria and repeated infection[11,12]. As such it is increasingly evident that stunting will not be resolved by improved nutritional intake or acute rehabilitation alone[13] but with parallel improvements in water quality, sanitation and hygiene (WASH) which act as a primary barrier to infection.

Recent cluster-randomised control trials have sought to investigate the effect of improved WASH, alone and in combination with nutrition supplementation, on child health. However different study designs and settings have for the most part failed to show consistent evidence for a reduction in diarrhoea or improvements in malnutrition indicators[14–19]. One possibility is that despite thorough design, interventions mainly focused on containing human excreta and did not consider (and conventionally have not considered) the role of animal faeces in domestic contamination and illness:[20] surprising given over 60% of infectious diseases in humans are caused by zoonotic pathogens[21]. Transmission pathways are not mutually exclusive, and inadequate separation of animals from the home environment may inevitably result in faecal-oral transmission through direct contact with animal faeces or contaminated soil, or faecal contamination of hands, food, objects or water sources[22–24]. Infants are also vulnerable to transmission routes specific to age-related behaviours,

including contaminated floors, where they crawl and directly or indirectly ingest faecal material[25–27]. As such, animal faecal contamination is a neglected factor potentially contributing to infection, diarrhoea and linear growth failure.

Infant *Campylobacter* infection and transmission

Previous studies have sought to understand the disease burden attributed to animal faeces which acts as a transmission vector via the faecal-oral pathway[20–22]. Key zoonotic pathogens related to infant infection, growth failure and EED include G*iardia*[28,29]*, enteroaggregative* and *enteropathogenic E. coli*[28–30], *Shigella*[31,32] and *Cryptosporidium*[33,34] which are transmitted across multiple pathways within the home and ingested through normal infant hand-to-mouth behaviour[25,35]. Among those pathogens of highest concern, *Campylobacter* consistently emerges as one of the key contributors to diarrhoea and malnutrition[31,32,34] and EED[28]. One of the most widespread infectious diseases, Campylobacteriosis is endemic across lower-income countries, especially in children[36] – responsible for 30,931 diarrhoeal-related deaths in 2015[37]. The infectious dose for Campylobacteriosis is low compared to other bacterial infections, with reported minimum values of around 500 CFU leading to infection in adults:[38,39] this value may also be lower for infants where immune systems are immature. Infection is acute and generally self-limiting: however while mean excretion is reported at around seven days[36], the bacteria has been isolated from faeces up to two weeks following infection[40,41]. Prolonged excretion may enhance transmission and incidence[42] and where it also affects the epithelial barrier[43] may contribute to gut mucosal damage and other EED-like abnormalities[44].

Large studies across many different low-income settings have attributed both asymptomatic and symptomatic *Campylobacter* infection with shorter length attainment of up to one centimetre[32,44] and with changes in EED clinical markers[43,45]. Thermophilic *C. jejuni* (~90%) and *C. coli* are the most commonly isolated *Campylobacter* species in diarrhoeal disease[46], and as part of the normal intestinal flora of birds, poultry represents one of the major sources

of transmission, contamination and infection[47]. An essential component of livelihoods and nutrition security, poultry ownership – particularly chickens – is ubiquitous across many low-income nations[48]. Largely free-ranging and dependent on scavenging, chickens frequently openly defecate inside the home and so infants are frequently exposed to, and often consume, chicken faeces and/or contaminated floor surface material during crawling or play[27,49,50]. As domestic floors are usually made of compacted soil, detection and removal of small poultry faeces is difficult and so Campylobacteriosis risk in crawling infants is high. Beyond six months of age critical developmental stages of weaning and crawling mean infection risk increases,[51] with obvious implications for short- and long-term growth and development. However, the evidence base describing the links between domestic animal ownership (particularly chickens), WASH facilities and use and infant nutritional status is limited to a few observational studies[27,52–55], which have not consistently measured *Campylobacter* carriage and/or infection. There is insufficient evidence to fully describe the extent to which infection is caused by exposure to domestic animals in low- and middle-income countries, and furthermore, if infant nutritional status affects whether infection is clinical or sub-clinical.

Study aims

Thus there is a need for further research which describes *Campylobacter* prevalence in young infants and the relationship to animal ownership and health ~~status~~outcomes, whilst ~~and~~ also ~~in relation to~~ considering ~~existing~~ household WASH facilities and use. Further data is also needed on infection and age-related transmission pathways, including domestic floors which are of high risk to this age group[56–58]. This ~~formative~~ small study aimed to provide formative evidence toward the prevailing hypothesis that infant health is negatively associated with stools positive for *Campylobacter* ~~infection~~ and exposure to domestic animals, ~~and~~ whilst not mitigated by WASH facilities. It aimed to ~~answer the following questions~~determine: i) ~~What is the prevalence of I~~infant *Campylobacter* prevalence in a sample of rural, subsistence households in Sidama zone, Ethiopia with domestic animals ii)

~~What is~~ Tthe relationship between both asymptomatic and symptomatic *Campylobacter* ~~infection~~ positive infants and anthropometric indices across households~~?~~ and iii) ~~What are some of the~~ R~~r~~isk factors and possible transmission pathways associated with infants positive for ~~infection?~~*Campylobacter*.

2. Methods

Country context and study sample

In Ethiopia, despite substantial recent reductions, linear growth failure affected more than a third of infants in 2016[59]. Ethiopia has one of the highest domestic animal densities per km^2 worldwide[60] and poultry are ubiquitous in rural households. ~~Whilst some~~Some research in Ethiopia has documented the proximity and exposure of infants to chickens and their faeces in regions[61] and the relationship with infant growth[54], and a few regional studies have associated *Campylobacter* infection with infant diarrhoea and malnutrition.[62–64]. However f~~f~~urther research is required in Ethiopia on the epidemiology of infant *Campylobacter* prevalence and infant health outcomes and the relationship to poultry ownership and WASH facilities. This small, formative study was conducted in ~~Sidama zone,~~ the Southern Nations, Nationalities, and Peoples' region (SNNPR), Sidama zone (regional subdivision), Ethiopia, as the geographical outreach area of the non-governmental organisation People in Need. The study took part in the month of June 2019 – the start of the region's rainy season. Two rural kebeles (neighbourhoods) were chosen from a woreda (zonal subdivision) which remained representative of typical rural livelihoods across Sidama ~~the~~ z~~Z~~one. A simple random sampling method was used to identify households fulfilling the eligibility criteria ~~with~~ of having an infant aged 10−18 months and owning free-range poultry. The random sample is described as follows. After communication with a government Health Extension Worker (HEW) local to each kebele, the team produced a sampling frame for both kebeles of all infants aged 10−18 months from households known by the HEW to own poultry. For both sampling frames, households were sequentially numbered on paper and using a simple

lottery method 17 and 18 infants were randomly ~~selected~~ drawn from the two kebele frames respectively for a total sample of 35 infants. Households were visited on a single occasion.

As this study was designed to provide formative evidence, a sample size calculation was not performed. Formative research is often conducted as part of the process of a larger study design and provides data for research teams to plan interventions or further data collection. Formative research is early phase data and is not powered to detect differences between groups. As such, this study results must be interpreted in this context, where it provided indicative data towards the hypothesis but was not sufficiently powered for conclusive evidence[65].

Survey and anthropometry

A ~~previously validated~~ survey previously validated in the region[50] assessed latrine type and use, handwashing practices and soap availability, domestic ~~livestock~~ animal ownership and ~~animal~~ husbandry practices and infant diarrhoeal prevalence and duration ~~of episodes~~. ~~Presence and evidence of use of a working latrine and handwashing station were also validated by direct observation.~~ To assess diarrhoea, caregivers were asked the frequency of loose or watery stools during the last day and over the past seven days. World Health Organisation (WHO) criteria was applied retrospectively, where diarrhoea is defined as at least three loose or watery stools within a 24-hour period[66]. Reported diarrhoea was later compared with the quality of stool samples, where all cases of reported diarrhoea matched visible diarrhoeal stool consistency. Presence and evidence of use of a working latrine and handwashing station were also validated by direct observation. After primary introductions with the caregivers and informed consent, a fieldworker completed the survey with translation from the HEW. Anthropometry measures were infant recumbent length (measured to the nearest 0.1 cm) and weight (measured to the nearest 100 g), taken by trained personnel following standard procedures[67] using a hanging Salter scale and a portable, fixed base length board.

Sample collection and transport

A day prior to ~~each~~ household visits, HEWs distributed sterile sample collection bags with a sterile scoop to households for faecal sample collection (Whirl-Pak® WPB01478WA, Sigma-Aldrich, UK). Caregivers were shown how to use the sterile scoop and seal the bags to minimise contamination, and were requested to collect a fresh faecal sample from their poultry and infant as close as possible to sample collection within 24 hours. Households were instructed to collect poultry samples from inside the home. During the study visit a third sample was collected from the floor surface inside the home. The infant's mother was asked to indicate the location the infant usually plays, and a researcher collected a sample of compacted floor surface (approximately 20 g) into another collection bag. All samples were transported in an insulated cool bag on ice to the laboratory at Hawassa University College of Medicine and Health Sciences within five hours. Upon arrival to the laboratory, samples were stored refrigerated (2−8°C) prior to analysis and plates were inoculated and incubated within two hours of arrival to the laboratory. ~~-~~ Plates were discarded safely following successful growth. Sample collection and transport methods echo similar methods in studies conducted in Ethiopia[64]. Thus each household sampling event (N=35) comprised three samples (poultry and infant faeces and floor surface). Due to damaged collection bags, three floor surface samples were discarded to give a total of 102 samples analysed for presumptive *Campylobacter* spp. Samples were numbered anonymously which linked the relevant household but removed all identifiers. ~~Upon arrival to the laboratory, samples were stored refrigerated (2−8°C) prior to analysis and discarded safely following successful growth.~~

Isolation of *Campylobacter* spp.

Presumptive thermotolerant *Campylobacter* spp. was isolated from fresh faecal samples from poultry, ~~from~~ infants ~~with and without diarrhoea~~ and ~~from~~ floor surface samples. Methods were briefly as follows. Aseptic techniques were followed and samples weighed

using sterile disposable weighing boats to 1 ± 0.05 g wet weight. Samples were then aliquoted into sterile plastic centrifuge tubes containing 9 mL of prepared sterile peptone water and vortexed well. For poultry faecal samples only, 100 µL of sample was pipetted into sterile tubes containing 900 µL of peptone water to prepare a 10-fold serial dilution up to 10^5 dilution. 100 µL of floor surface and infant faecal samples and poultry faecal sample dilutions of orders 10^1, 10^3 and 10^5 were drop plated on pre-labelled plates and spread using disposable L-shaped spreaders. Blood-free chromogenic CHROMagar™ Campylobacter media (CHROMagar™, France) was used for the selective detection and differentiation of presumptive thermotolerant *Campylobacter*, prepared and used according to manufacturer instructions[68]. Inoculated plates were allowed to dry under a laminar flow for approximately five minutes as per manufacturer instructions, inverted and stacked into anaerobic jars and incubated at 42°C for 48 hours under microaerophilic conditions. CampyGen™ 2.5 L sachets (Thermo Scientific™, UK) were used to obtain a hydrogen-free microaerophilic atmosphere of approximately 5% O_2, 10% CO_2 and 85% N_2, suitable for the growth of *Campylobacter* spp.

Identification of *Campylobacter* spp.

After 48 hours, presumptive *C. jejuni*, *C. coli* and *C. lari* appear on the chromogenic agar as intense red coloured colonies on a translucent base. Other non-target microorganisms are inhibited (i.e. small, blue colour or absent colonies[68]) and~~with~~ high specificity and sensitivity versus other media is well demonstrated[69–71]. Quality control and preparation of the medium was tested by isolating the ATCC® strain *C. jejuni* (33291) under representative conditions at Cranfield University prior to fieldwork. Blank samples with no growth confirmed no external contamination in all batches. ~~This fieldwork originally aimed to use quantitative PCR and sequencing techniques for the detection of and quantification of *Campylobacter* to support culture data, as well as to assess the performance characteristics of qPCR and culture to measure *Campylobacter* in faecal samples in a low-income setting. However, prolonged and~~

~~repeated power cuts in the field meant that this proved impossible. The challenges encountered during fieldwork will be reported separately (Budge S. et al, in preparation).~~

Ethics

At the start of each household visit, the study was introduced by the field team and HEW and informed consent was described to the caregiver in their first language of Amharic or Sidamo. Fieldworkers tested the caregivers' understanding of consent by asking them questions regarding the study and~~or~~ the consent process, and explained all data was anonymised. As most adult caregivers were illiterate, oral consent and assent for their infant was recorded. The survey was written in English, translated to Amharic by the field team and verbally translated into Sidamo by a HEW. The study protocol was approved by two institutional review boards: Cranfield University Research Ethics Committee (CURES/7774/2019) and Hawassa University College of Medicine and Health Sciences (IRB/222/11).

Statistical analysis

Analyses were performed at the household level. Plates were visually inspected for presumptive *Campylobacter* spp. and recorded as growth or non-growth and the prevalence (as percentage) of positive poultry, infants and floor surfaces was calculated. Whilst the presence of *Campylobacter* does not necessarily indicate active infection, for the purpose of analysis, ~~infants with faeces positive for~~samples with presumptive *Campylobacter* growth were classified as '~~infected~~positive' or with no growth as 'negative'. ~~Infection~~ Positive infant faecal samples were~~was~~ then ~~further~~ described as symptomatic (the positive stool sample was diarrhoeal), or asymptomatic (~~without~~ the stool sample was not diarrhoeal). Z scores were calculated for length-for-age and weight-for-length (LAZ and WLZ respectively) using the WHO 2006 Child Growth Standards[72]. Z-scores were categorised into stunting and wasting using the standard cut-off value less than −2 standard deviations of the reference[72]. Anonymised household survey data were entered into Microsoft Excel, coded for descriptive

analysis and further analysed using SPSS (version 22.0, IBM, New York). Simple frequency distribution tests described survey response data, anthropometric data and *Campylobacter* prevalence. Fisher's exact test for independence tested associations between variables for the small sample size (5% significance). Results with significant p-values from the Fisher's exact test reported odds ratio (OR) risk estimates with corresponding 95% confidence intervals (CI).

3. Results

Survey and anthropometric data

Data were collected from all 35 households identified in the sampling frame. Results from the survey and anthropometric data are shown in Table 1. Average infant age was 15 months. Almost a third (31.4%) of infants had experienced diarrhoea within the past 7 days with an average duration of 3.1 days. 88.6% of households owned a latrine, most of which were improved pit latrines with a slab (82.9%). Less than half (40.0%) of households had some form of handwashing facility available (including a simple basin and jug) and half (51.4%) owned soap. Aside from poultry ownership, cattle was the second most common form of animal husbandry (n=19, 54.3%). Regarding animal husbandry practices 97.1% of households reported that during the day their animals shared the same living space as the family, and 91.4% during the night. Mean WLZ score was -0.61 (range -2.14−0.64, SE 0.15) and mean LAZ score was -0.81 (range -2.53−0.94, SE 0.19). Overall, five infants (14.3%) were classified as wasted (WLZ <-2 SD), eleven (31.4%) as stunted (LAZ <-2 SD) and four infants both wasted and stunted (11.4%, WLZ and LAZ <-2 SD). Of those infants classified as wasted (n=5), all had experienced diarrhoea within the past seven days ($p<0.001$; OR 1.83, 95% CI 1.07−3.14). Diarrhoeal prevalence was not significantly related to stunting ($p=0.709$).

Table 1. Infant and household characteristics (N=35) including animal husbandry practices and anthropometric indicators, Sidama zone, Ethiopia.

Household characteristic	n	~~% of total~~Average or percent (%) of total
Infant sex		
Male	19	54.3%
Female	16	45.7%
Average age (months)	~~15~~	15
Diarrhoea during the last 7 days	11	31.4%
Average duration of diarrhoea (days)	~~3.7~~	3.7
Household owns a latrine	31	88.6%
Household latrine type		
Open defecation (no latrine)	1	2.9%
Use neighbour's toilet (no latrine)	3	8.6%
Pit latrine without slab	2	5.7%
Pit latrine with slab	29	82.9%
Household has a handwashing facility	14	40.0%
Household has soap available	18	51.4%
Household domestic livestock ownership		
Chickens	35	100%
Cattle	19	54.3%
Goats	11	31.4%
Donkey(s)	2	5.7%
Livestock practices during the day:		
Live outside	35	100.0%
Live inside in the same room as the family	34	97.1%
Live inside in a separate room to the family	1	2.9%
Livestock practices during the night:		
Live inside in the same room as the family	32	91.4%
Live inside in a separate room to the family	3	8.6%
**Nutrition indicator**	**n**	**Percent (%) of total**
Weight-for-length (WLZ)		
-2 to -3 SD (wasted)	5	14.3%
Length-for-age (LAZ)		
-2 to -3 SD (stunted)	11	31.4%
WLZ and LAZ		
-2 to -3 SD (stunted and wasted)	4	11.4%

Campylobacter ~~infection~~ prevalence and correlation with infant health measures

The following sections describe the relationships between survey variables, prevalence of presumptive *Campylobacter* ~~infection~~ and infant health outcomes. A total of 102 samples from poultry, infants and floor surface were cultured for *Campylobacter* spp. Overall, *Campylobacter* was recovered from 48.6% (n=17) of 35 infant faecal samples, 68.6% (n=24) of 35 poultry faecal samples and 65.6% (n=21) of 32 floor surface samples. Differences in the prevalence of ~~infection~~ positive samples which ~~was~~ were symptomatic (a diarrhoeal stool sample) and asymptomatic (non-diarrhoeal stool, 'carriers') ~~were~~ was seen among positive infants presenting with diarrhoeal stools (n=10, 58.8%) versus without diarrhoea (n=7, 41.2%) (p<0.001). Furthermore, ~~all infants who were~~infant who were wasted (low weight-for-length) -versus not wasted were compared for *Campylobacter* prevalence. Those wasted were more likely to test ~~wasted (low weight for length) were~~ positive for *Campylobacter* (p=0.019; OR 1.41, 95% CI 1.04−1.92). Wasted infants thus appeared to have a 1.83 times the odds of diarrhoea and 1.41 times of a sample positive for *Campylobacter* versus those not wasted. However, diarrhoea was not associated with infant stunting (p=0.709), nor was ~~infection~~ *Campylobacter* prevalence (p=0.725).

Risk factors and transmission pathways related to infant _Campylobacter_ ~~infection~~ prevalence

Further analysis using correlation explored the relationship between potential risk factors and transmission pathways to infant stools positive for *Campylobacter* ~~infection~~. Considering associated risk factors, animal husbandry practices of keeping animals inside during the day and night (as a composite variable) was strongly correlated with increased odds of infants positive for ~~infection~~ *Campylobacter* (p=0.027, OR 3.5, 95% CI 1.31–8.77). Owning donkeys or goats showed no association (p=0.229 and p=0.546 respectively), but owning cattle was significantly associated with increased odds, although with high uncertainty of effect (p=0.018, OR 6.5, 95% CI 1.47−28.90). Poultry faeces positive for *Campylobacter* showed significant correlation with infant *Campylobacter* ~~infection~~ (p<0.001, OR 1.34, 95%CI

1.21−1.69). However, owning a latrine, different types of latrine, owning a handwashing facility and ownership of soap were all not correlated~~ with infant infection~~ (all p>0.5).~~,~~ ~~showing no mitigating effect of improved WASH facilities.~~ Considering potential transmission pathways, positive floor samples showed a significant association, although again with high uncertainty of effect (p=0.023, OR 7.0, 95% CI 1.5−23.4). Positive poultry faeces and positive floor samples were also highly correlated (p=0.013; OR 2.68. 95% CI 1.64−12.62).~~,~~ ~~highlighting the importance of contaminated floors as a transmission route for infant pathogen ingestion.~~ ~~Fig 1 describes~~ The associations between ~~these~~ risk factors and transmission pathways in relation to infant health outcomes are detailed in Table 2. Fig. 1 also illustrates these associations whereby~~.~~ t~~T~~he dotted lines describe the main transmission pathways to an infant stool positive for *Campylobacter*.

Table 2. Odds ratios (OR) for exposure measures predicting an infant stool positive for *Campylobacter*, with corresponding confidence intervals (CI) and p values (significance < 0.05).

**Variable**	**OR**	**95% CI**	**P value**
Infant wasting (WLZ <−2 SD)	1.41	1.04−1.92	0.019
Positive poultry faeces	1.34	1.21−1.69	<0.001
Keeping animals inside (day and night)	3.50	1.31–8.77	0.027
Owning cattle	6.50	1.47−28.90	0.018
Positive floor sample	7.00	1.50−23.40	0.023

Fig 1. Diagram exploring pathways between variables that predict infant ~~infection~~ stools positive for ~~with~~ Campylobacter *and the relationship with health outcomes. Dotted lines demonstrate the hypothesised pathway linking poultry ownership,* Campylobacter *prevalence ~~infection~~ and health outcomes via clinical or subclinical disease.~~-~~ 'Symptomatic'*

infection refers to infants positive for Campylobacter who also had a diarrhoeal stool. P values <0.05 were deemed significant. This diagram is expanded in Fig 2. CB, Campylobacter; WLZ, weight-for-length; LAZ, length-for-age.

4. Discussion

Results from this small cross-sectional study suggest that in these rural Sidamo households raising free-range domestic poultry, the prevalence of infants testing positive for ~~infant~~ *Campylobacter* spp. ~~infection~~ is high~~ly prevalent~~. With presumptive *Campylobacter*~~ present i~~ isolated in almost half of infant stools, results mirror high prevalence found in similar age infants in Zimbabwe (32.3%)[73], Mexico (66.0%)[42], Madagascar (43.3%)[74] and across eight low-resource settings where 84.9% of infants had at least one positive faecal sample by one year of age[45]. The high prevalence in this study may be due to sample collection during the rainy season where pooled water inside the home facilitates the spread of faecal bacteria: however other studies have found constant high prevalence not affected by seasonality[44,75]. In this study, 58.8% of the 17 infants positive for *Campylobacter* were symptomatic with ~~diarrhoea~~ diarrhoeal stools. With~~With~~ an average *Campylobacter* excretion ~~an average duration~~ of seven days[36] (and reported protracted excretion of more than 14 days[40]) ~~it is possible that diarrhoea~~ this may lend support that current diarrhoea in these infants was from Campylobacteriosis. ~~Other studies have also reported an episode average of 3.8 days in infants [39], similar to the average length reported here~~ Studies in northern Ethiopia[62,63] and in the same zone as this study[64] suggest *Campylobacte*r is a major regional cause of diarrhoea ~~. Of those~~Comparing infants who were ~~measured as~~ wasted (n=5) versus those non-wasted, wasting was correlated with ~~all were positive for~~ positive *Campylobacter* prevalence~~ and experienced~~ and diarrhoeal stools. ~~diarrhoea~~. ~~It is thus possible that here symptomatic infection was a causal factor for acute malnutrition~~ Campylobacteriosis may have contributed to these outcomes, but it is likely other coexisting infections also contributed[32,76].

Whilst in early infancy infection may produce clinical symptoms and affect short-term weight, repeated enteropathogens colonisation may contribute to the development of EED~~ as an asymptomatic, subclinical disease. ~~.~~In EED, reduced intestinal function and nutrient absorption affect linear growth in a cyclic interaction [69,70].~~ Although this study was not able to collect biological measures of EED, 41.2% (n=7) of positive infant stools were asymptomatic (~~that is, infants were~~non-diarrhoeal stools~~ carriers~~). This supports findings from the MAL-ED study ~~that~~ where subclinical infection ~~from enteropathogens were~~ was more strongly related to growth failure than overt diarrhoea[12]. Although ~~infection~~ positive stools showed no significant correlation with stunting, this may be ~~a reflection of the~~ partly due to the small sample size. F~~: f~~urthermore, research suggests that ~~the effect on~~ growth shortfalls resulting from early ~~infant~~ exposure to *Campylobacter* manifests later in infancy[31]. Studies have associated cohabiting with poultry with reduced length-for-age[53,77] and others have shown that infants who frequently test positive for *Campylobacter* have lower LAZ scores at 24 months of age, which ~~strongly correlated~~ had a stronger correlation with subclinical infection, or *Campylobacter* carriage[31,44]. Other studies have also demonstrated a relationship between poultry ownership and lower WAZ but not lower LAZ[53], ~~but which does~~ suggesting both ~~an~~ acute and chronic effects on ~~development~~ health. ~~Further importance~~ Other significance lies in the overlap between wasting and stunting among infants in this group (p=0.026), supporting ~~discussions~~ evidence that ~~different~~ the two forms of malnutrition can, ~~(~~and often do,~~)~~ coexist in the same infant[78], that they ~~and that wasting and stunting~~ may share common causal factors of repeated carriage and/or infection[79].

This study aimed to further describe the relationship between domestic animal ownership and infant ~~infection~~ pathogen prevalence and growth, where~~by~~ free-roaming domestic animals may contribute to contamination of the home environment with pathogenic bacteria. Indeed in this study, households were instructed to collect poultry samples from indoors and only two collected samples from outdoors, highlighting the ubiquity of poultry faeces inside

the home. Infection is possibly transmitted to infants via age-specific behaviours and pathways. In this study, the significant risk factors that correlated with positive infant ~~*Campylobacter*~~stools~~ infection~~ were specific animal husbandry practices of keeping animals inside during the day and night (~~in each instance in~~ ubiquitously in the same room as the family), owning cattle, positive domestic floor samples and positive poultry faeces. ~~Although~~ ~~a~~The analysis showed some uncertainty of effect and the small sample size may reduce the validity of findings, however the results do highlight specific risk factors to infants, including ~~the importance of~~ contaminated domestic floors~~ contamination~~ as a potentially important transmission pathway. ~~This is supported by L~~Longitudinal data from the MAL-ED team ~~which~~ showed the effect of *Campylobacter* infection on growth is ~~largely~~ related to age – highlighting an increased level of risk as infants start to crawl[31]. Whilst this study did not capture hand-to-mouth contact events, previous research by this team ~~in similar households~~ in the same geographical area recorded infants mouthed their own hands or those of their caregiver a mean 31 and 21 times respectively over one ~~-~~hour, which ~~in the majority of instances~~ were often ~~both~~ visibly dirty (90.0% and 86.0% respectively)[50]. In the same study 35.0% of infants directly ingested floor surface material and poultry faeces was directly ingested by two infants (10.0%)[50]. Other studies have also recorded infants frequently ingesting poultry faeces from the floor during normal exploratory play[27,80,81].

Other factors not measured in this study, such as contaminated hands, food (particularly milk) and drinking water~~,~~ ~~would likely~~may account for the remaining sources of and transmission pathways to infant infection. Although a fastidious organism, *Campylobacter* is widespread in the environment, transmitted particularly through contaminated groundwater and stored drinking water[46], surviving for several days in an ambient environment[49]. Consequently research has suggested that in households where poultry are free-roaming, even with good water supply it is unlikely handwashing will effectively interrupt transmission[49]. *Campylobacter* transmission is also increased when WASH facilities are poor:[36] similar cross-sectional studies in Ethiopia also found higher *Campylobacter*

prevalence in households without clean water and which had direct contact with chickens[82,83]. In this study latrine ownership and type (improved or not), ownership of handwashing facilities and soap were not correlated with ~~a reduction in~~ stool samples negative for *Campylobacter*, perhaps suggesting that~~e~~ ~~inability of~~ simply providing WASH facilities will not~~to~~ prevent transmission and infection. However it is possible facilities are also not used, particularly by children, which remains a limitation. In rural communities it can be difficult to assess and accurately report the use of latrines and soap for handwashing. Whilst in this study the visual inspection of latrines suggested they were all used, soap ownership would often be reported but not seen. Regardless, it seems logical that when sharing living spaces so closely, domestic animals contribute to infection from zoonoses and widespread contamination ~~across~~ of multiple pathways ~~by way of open defecation~~. There are intrinsic and inseparable connections between these various transmission pathways. This is illustrated in Fig 2~~.~~, ~~Incorporating the original 'F diagram' with its principal focus on human excreta [77], Fig 2 combines findings from this study to map out~~which illustrates ~~the~~ causal pathways to poor infant health outcomes when animal faecal contamination and age-specific infant behaviours are not considered as important risk factors.

Fig 2. The hypothesised pathways by which domestic poultry ownership contributes to acute and chronic infant malnutrition via infection from, and transmission of, Campylobacter. The thicker part of the diagram illustrates the hypothesised relationship with environmental enteric dysfunction (EED). The dotted part of the diagram to the upper right constitutes the original 'F diagram', representing other transmission pathways by which infants are exposed to Campylobacter. Adapted alongside a previously published diagram[9] and the 'F published by Wagner, E. and Lanoix, J., 1958[84].

The validity and broader applicability of findings from this study are mostly limited by the small sample size which may affect data validity and generalisability of the results. The single time point of testing in this formative research and the cross-sectional study design

~~also~~ prevent determining causality. However, the results are emphasised as formative evidence, and support emerging hypotheses which associate free-range poultry ownership, household contamination and infant infection with ~~linear growth faltering~~ undernutrition. Although this study intentionally sampled households who owned poultry, the risk of transmission may actually be greater than estimated as free-range chickens from neighbouring households may also increase contamination. ~~On the other hand,~~ Also, faecal samples from other domestic animals which also harbour *Campylobacter*, such as cattle[46], were not sampled. ~~which may have contributed to contamination and infection.~~. ~~Similarly,~~ On the other hand there was no evaluation of the prevalence of other pathogenic or parasitic organisms, so ~~and~~ it is ~~therefore~~ not certain that the presumptive *Campylobacter* isolated in samples was the definite cause of wasting and/or diarrhoeal prevalence seen here. ~~Only~~ A~~a~~ few studies have reported mixed infections of *Campylobacter* and viral pathogens and their associations with infant morbiditie~~y~~s[32,85]. This presence of *Campylobacter* alongside the carriage of multiple pathogens may correlate as a proxy for infants with greater overall levels of exposure to enteric pathogens in their environment; this in turn may associate with those with poor growth and/or wasting.

Lastly, the use of culture-based method alone ~~(and without sample pre-enrichment)~~ holds limitations: firstly due to changes in *Campylobacter* cell physiology and loss of viability between sample deposition, collection, transport and plating (whereby cells enter the viable but non-cultivable [VBNC] state). This may have underestimated the true prevalence. ~~and by~~ On the other hand, culture holds limited sensitivity and high rates of false detection;[86] ~~.~~ whilst there is evidence for good specificity of the agar in comparison and evaluation studies, there is no certainty of the rate of false positives in this study. ~~Secondly,~~ Lastly, whilst the culture media shows high specificity, it was not possible to differentiate between or quantify different *Campylobacter* species. The parallel use of qPCR alone or PCR with ELISA methods would enhance culture-based findings[31,87].

Conclusion

This formative study adds further preliminary evidence to the body of research documenting infant *Campylobacter* carriage and infection in households rearing free-range poultry~~-rearing households~~. In these households, increased wasting and diarrhoea was seen in ~~in infected~~ infants positive for presumptive *Campylobacter*~~ highlights the potential impact of acute infection~~. Repeated symptomatic infection and low weight ~~weight loss~~ may mean infants risk entering a spiral of ~~lower weight gain~~ weight loss and subsequent growth deficits. Alternatively, frequent carriage, or asymptomatic infection, and a high prevalence of stunting (although not correlated) suggest a longer-term impact of exposure to *Campylobacter* that may operate through EED~~-related mechanisms~~. The time frame for when, and thresholds at which repeated *Campylobacter* infection becomes subclinical, contributes to the development of EED and ~~also~~ affects growth are important remaining questions ~~for research~~ which a larger prospective cohort might address.

More broadly, this study also contributes to discussions around general WASH facilities and use, living conditions and the impact on reducing pathogen transmission. Where contaminated domestic floors are a risk factor for pathogen transmission to infants[56,88] and WASH facilities also appear have little effect in mitigating transmission, ~~results~~ this emphasises the high thresholds of hygiene and ~~development~~ living conditions necessary to improve infant health~~ and reduce morbidities~~. While improvements to ~~water quantity, quality, and handwashing~~basic WASH usually included in interventions may address some secondary pathogen transmission routes, a remaining burden of infection may be expected when animals share the living space. An extensive, multifaceted approach to improve infant health will require not only improved WASH facilities, but working with communities to adapt current animal husbandry practices, encourage the safe handling and disposal of both animal and adult/infant faeces, safe preparation and storage of food, handwashing with soap after animal/faecal contact and education on the health risks of infant exposure. These multiple, concurrent needs form the rationale for the recent push toward 'transformative

WASH'[89] or 'WASH++'[90]. ~~Certainly, future~~Future research ~~objectives~~ in the WASH sector must ~~be much bolder and aim to~~ develop and test transformative WASH interventions ~~that are radically more effective in reducing faecal contamination in the home environment~~ if we are to achieve the high hygiene thresholds that support optimal infant growth.

Acknowledgements

Sophie Budge wrote the manuscript. All other authors assisted in assessing both the paper quality and contributed to the writing and review of the manuscript. The authors wish to thank all of the People in Need team at the Hawassa office who assisted in data collection, logistics and planning. They would also like to thank Sara and Abebe at Hawassa University College of Medicine and Health Sciences and the wider faculty staff who gave their space and time. Finally the authors thank all of the study participants who kindly welcomed us into their homes and offered their time.

Declaration of interest

All authors have no conflict of interest to declare.

Data access

All data created during this research is openly available from the Cranfield Online Research Data at 10.17862/cranfield.rd.9907385.

Supporting information legends

S1 Checklist: STROBE Checklist

Survey 1.

References

1. Liu L, Oza S, Hogan D, et al. Global, regional, and national causes of under-5 mortality in 2000–15: an updated systematic analysis with implications for the

Sustainable Development Goals. *Lancet*. 2016;388(10063):3027-3035. doi:10.1016/S0140-6736(16)31593-8

2. Fischer Walker CL, Perin J, Aryee MJ, Boschi-Pinto C, Black RE. Diarrhea incidence in low- and middle-income countries in 1990 and 2010: A systematic review. *BMC Public Health*. 2012;6(3):E230-E231. doi:10.1186/1471-2458-12-220
3. Danaei G, Andrews K, Sudfeld C et al. Risk Factors for Childhood Stunting in 137 Developing Countries: A Comparative Risk Assessment Analysis at Global, Regional, and Country Levels. *PLoS Med*. 2016;13(11):1–18.
4. Troeger C, Colombara D V., Rao PC, et al. Global disability-adjusted life-year estimates of long-term health burden and undernutrition attributable to diarrhoeal diseases in children younger than 5 years. *Lancet Glob Heal*. 2018;6(3):e255-e269. doi:10.1016/S2214-109X(18)30045-7
5. Guerrant RL, Oriá RB, Moore SR, Oriá MOB, Lima AAAM. Malnutrition as an enteric infectious disease with long-term effects on child development. *Nutr Rev*. 2008;66(9):487-505. doi:10.1111/j.1753-4887.2008.00082.x
6. Bhutta Z, Ahmed T, Black R et al. What works? Interventions for maternal and child undernutrition and survival. *Lancet*. 2008;371(9610):417–440.
7. Lunn P, Northrop-Clewes C, Downes R. Intestinal permeability, mucosal injury, and growth faltering in Gambian infants. *Lancet*. 1991;338(8772):907–910.
8. Keusch GT, Denno DM, Black RE, et al. Environmental enteric dysfunction: Pathogenesis, diagnosis, and clinical consequences. *Clin Infect Dis*. 2014;59(S4):S207–S212. doi:10.1093/cid/ciu485
9. Budge S, Parker AH, Hutchings PT, Garbutt C. Environmental enteric dysfunction and child stunting. *Nutr Rev*. 2019;77(4):240–253. doi:10.1093/nutrit/nuy068
10. Owino V, Ahmed T, Freemark M et al. Environmental Enteric Dysfunction and Growth Failure/Stunting in Global Child Health. *Pediatrics*. 2016;138(6):e20160641–e20160641.
11. Ahmed T, Auble D, Berkley J et al. An evolving perspective about the origins of

childhood undernutrition and nutritional interventions that includes the gut microbiome. *Ann N Y Acad Sci*. 2014;1332(1):22–38. doi:10.1111/nyas.12487

12. MAL-ED Network Investigators. Relationship between growth and illness, enteropathogens and dietary intakes in the first 2 years of life: Findings from the MAL-ED birth cohort study. *BMJ Glob Heal*. 2017;2(4):e000370. doi:10.1136/bmjgh-2017-000370

13. Dewey K, Adu-Afarwuah S. Systematic review of the efficacy and effectiveness of complementary feeding interventions in developing countries. *Mater Child Nutr*. 2008;4(S1):24–85. doi:10.1111/j.1740-8709.2007.00124.x

14. Luby SP, Rahman M, Arnold BF, et al. Effects of water quality, sanitation, handwashing, and nutritional interventions on diarrhoea and child growth in rural Bangladesh: a cluster randomised controlled trial. *Lancet Glob Heal*. 2018;6(3):e302-e315. doi:10.1016/S2214-109X(17)30490-4

15. Null C, Stewart CP, Pickering AJ, et al. Effects of water quality, sanitation, handwashing, and nutritional interventions on diarrhoea and child growth in rural Kenya: A cluster-randomised controlled trial. *Lancet Glob Heal*. 2018;6(3):e316–e329. doi:https://doi.org/10.1016/S2214-109X(18)30005-6

16. Humphrey JH, Mbuya MNN, Ntozini R, et al. Independent and combined effects of improved water, sanitation, and hygiene, and improved complementary feeding, on child stunting and anaemia in rural Zimbabwe: a cluster-randomised trial. *Lancet Glob Heal*. 2019;7(1):e132–e147. doi:10.1016/S2214-109X(18)30374-7

17. Clasen T, Boisson S, Routray P, et al. Effectiveness of a rural sanitation programme on diarrhoea, soil-transmitted helminth infection, and child malnutrition in Odisha, India: A cluster-randomised trial. *Lancet Glob Heal*. 2014;2(11):e645-e653. doi:10.1016/S2214-109X(14)70307-9

18. Cameron L, Olivia S, Shah M. Scaling up sanitation: Evidence from an RCT in Indonesia. *J Dev Econ*. 2019;138:1-16. doi:10.1016/j.jdeveco.2018.12.001

19. Patil SR, Arnold BF, Salvatore AL, et al. The effect of India's total sanitation campaign

on defecation behaviors and child health in rural Madhya Pradesh: A cluster randomized controlled trial. *PLoS Med*. 2015;11(8):e1001709. doi:10.1371/journal.pmed.1001709

20. Delahoy MJ, Wodnik B, McAliley L, et al. Pathogens transmitted in animal feces in low- and middle-income countries. *Int J Hyg Environ Health*. 2018;221(4):661-676. doi:10.1016/j.ijheh.2018.03.005

21. Taylor LH, Latham SM, Woolhouse MEJ. Risk factors for human disease emergence. *Philos Trans R Soc B Biol Sci*. 2001;35(1411):983–989. doi:10.1098/rstb.2001.0888

22. Penakalapati G, Swarthout J, Delahoy M et al. Exposure to Animal Feces and Human Health: A Systematic Review and Proposed Research Priorities. *Envir Sci Technol*. 2017;51(20):11537–11552. doi:10.1021/acs.est.7b02811

23. Baker KK, Senesac R, Sewell D, Sen Gupta A, Cumming O, Mumma J. Fecal Fingerprints of Enteric Pathogen Contamination in Public Environments of Kisumu, Kenya, Associated with Human Sanitation Conditions and Domestic Animals. *Environ Sci Technol*. 2018;52(18):10263-10274. doi:10.1021/acs.est.8b01528

24. Ercumen A, Pickering AJ, Kwong LH, et al. Animal Feces Contribute to Domestic Fecal Contamination: Evidence from E. coli Measured in Water, Hands, Food, Flies, and Soil in Bangladesh. *Environ Sci Technol*. 2017;51(15):8725-8734. doi:10.1021/acs.est.7b01710

25. Morita T, Perin J, Oldja L et al. Mouthing of Soil Contaminated Objects is Associated with Environmental Enteropathy in Young Children. *Trop Med Int Heal*. 2017;22(6):670–678. doi:10.1111/tmi.12869

26. Mattioli MCM, Davis J, Boehm AB. Hand-to-mouth contacts result in greater ingestion of feces than dietary water consumption in Tanzania: A quantitative fecal exposure assessment model. *Environ Sci Technol*. 2015;49(3):1912-1920. doi:10.1021/es505555f

27. Ngure F, Humphrey J, Mbuya M et al. Formative research on hygiene behaviors and geophagy among infants and young children and implications of exposure to fecal

bacteria. *Am J Trop Med Hyg*. 2013;89(4):709–716. doi:10.4269/ajtmh.12-0568

28. George C, Burrowes V, Perin J et al. Enteric Infections in Young Children are Associated with Environmental Enteropathy and Impaired Growth. *Trop Med Int Heal*. 2017;12(10):3218–3221.

29. Kosek M et al. Causal Pathways from Enteropathogens to Environmental Enteropathy: Findings from the MAL-ED Birth Cohort Study. *EBio Med*. 2017;18:109–117. doi:10.1016/j.ebiom.2017.02.024

30. Lima AAM, Soares AM, Filho JQS, et al. Enteroaggregative Escherichia coli Subclinical Infection and Coinfections and Impaired Child Growth in the MAL-ED Cohort Study. *J Pediatr Gastroenterol Nutr*. 2018;66(2):325-333. doi:10.1097/MPG.0000000000001717

31. Rogawski ET, Liu J, Platts-Mills JA, et al. Use of quantitative molecular diagnostic methods to investigate the effect of enteropathogen infections on linear growth in children in low-resource settings: longitudinal analysis of results from the MAL-ED cohort study. *Lancet Glob Heal*. 2018;6(12):e1319-e1328. doi:10.1016/S2214-109X(18)30351-6

32. Platts-Mills JA, Taniuchi M, Uddin MJ, et al. Association between enteropathogens and malnutrition in children aged 6-23 mo in Bangladesh: A case-control study. *Am J Clin Nutr*. 2017;105(5):1132–1138. doi:10.3945/ajcn.116.138800

33. Checkley W, Epstein LD, Gilman RH, Black RE, Cabrera L, Sterling CR. Effects of Cryptosporidium parvum infection in Peruvian children: Growth faltering and subsequent catch-up growth. *Am J Epidemiol*. 1998;148(5):497-506. doi:10.1093/oxfordjournals.aje.a009675

34. Kotloff K, Nataro J, Blackwelder W et al. Burden and aetiology of diarrhoeal disease in infants and young children in developing countries (the Global Enteric Multicenter Study, GEMS): a prospective, case-control study. *Lancet*. 2013;382(9888):209–222. doi:10.1016/S0140-6736(13)60844-2

35. Perin J, Thomas A, Oldja L et al. Geophagy Is Associated with Growth Faltering in

Children in Rural Bangladesh. *J Pediatr*. 2016;178:34–39.
36. Coker AO, Isokpehi RD, Thomas BN, Amisu KO, Larry Obi C. Human campylobacteriosis in developing countries. *Emerg Infect Dis*. 2002;8(3):237-243. doi:10.3201/eid0803.010233
37. Troeger C, Forouzanfar M, Rao PC, et al. Estimates of global, regional, and national morbidity, mortality, and aetiologies of diarrhoeal diseases: a systematic analysis for the Global Burden of Disease Study 2015. *Lancet Infect Dis*. 2017;17(9):909-948. doi:10.1016/S1473-3099(17)30276-1
38. Hara-Kudo Y, Takatori K. Contamination level and ingestion dose of foodborne pathogens associated with infections. *Epidemiol Infect*. 2011;139(10):1505-1510. doi:10.1017/S095026881000292X
39. Black RE, Levine MM, Clements M Lou, Hughes TP, Blaser MJ, Black RE. Experimental campylobacter jejuni infection in humans. *J Infect Dis*. 1988;157(3):472-479. doi:10.1093/infdis/157.3.472
40. Rao MR, Naficy AB, Savarino SJ, et al. Pathogenicity and convalescent excretion of Campylobacter in rural Egyptian children. *Am J Epidemiol*. 2001;154(2):166-173. doi:10.1093/aje/154.2.166
41. Mølbak K, Højlyng N, Gaarslev K. High prevalence of campylobacter excretors among Liberian children related to environmental conditions. *Epidemiol Infect*. 1988;100(2):227–237. doi:10.1017/S0950268800067364
42. Calva JJ, Lopez-Vidal AB, Ruiz-Palacios GM, Ramos A, Bojalil R. Cohort study of intestinal infection with campylobacter in Mexican children. *Lancet*. 1988;1(8584):503-506. doi:10.1016/S0140-6736(88)91297-4
43. Chen ML, Ge Z, Fox JG, Schauer DB. Disruption of tight junctions and induction of proinflammatory cytokine responses in colonic epithelial cells by Campylobacter jejuni. *Infect Immun*. 2006;74(12):6581-6589. doi:10.1128/IAI.00958-06
44. Lee G, Pan W, Penataro Yori P, et al. Symptomatic and Asymptomatic Campylobacter Infections Associated with Reduced Growth in Peruvian Children.

PLoS Neglected Trop Dis. 2013;7(1):e2036. doi:10.1371/journal.pntd.0002036

45. Amour C, Gratz J, Mduma ER, et al. Epidemiology and Impact of Campylobacter Infection in Children in 8 Low-Resource Settings: Results from the MAL-ED Study. *Clin Infect Dis*. 2016;63(9):1171-1179. doi:10.1093/cid/ciw542
46. Kaakoush NO, Castaño-Rodríguez N, Mitchell HM, Man SM. Global epidemiology of campylobacter infection. *Clin Microbiol Rev*. 2015;28(3):687-720. doi:10.1128/CMR.00006-15
47. Janssen R, Krogfelt KA, Cawthraw SA, Van Pelt W, Wagenaar JA, Owen RJ. Host-pathogen interactions in Campylobacter infections: The host perspective. *Clin Microbiol Rev*. 2008;21(3):505-518. doi:10.1128/CMR.00055-07
48. FAO. *Decision Tools for Family Poultry Development. FAO Animal Production and Health Guidelines No. 16*. Rome, Italy; 2014.
49. Marquis G, Ventura G, Gilman R et al. Fecal contamination of shanty town toddlers in households with non-corralled poultry, Lima, Peru. *Am J Public Heal*. 1990;80(2):146–149. doi:10.2105/AJPH.80.2.146
50. Budge S, Hutchings P, Parker A, et al. Do domestic animals contribute to bacterial contamination of infant transmission pathways? Formative evidence from rural Ethiopia. *J Water Health*. 2019;17(5). doi:10.2166/wh.2019.224
51. Samuel SO, Aboderin AO, Akanbi AA, Adegboro B, Smith SI, Coker AO. Campylobacter enteritis in Ilorin, Nigeria. *East Afr Med J*. 2006;83(9):478-484.
52. George C, Oldja L, Biswas S et al. Fecal markers of environmental enteropathy are associated with animal exposure and caregiver hygiene in Bangladesh. *Am J Trop Med Hyg*. 2015;93(2):269-275. doi:10.4269/ajtmh.14-0694
53. Gelli A, Headey D, Becquey E, et al. Poultry husbandry, water, sanitation, and hygiene practices, and child anthropometry in rural Burkina Faso. *Matern Child Nutr*. 2019;e12818. doi:10.1111/mcn.12818
54. Headey D, Hirvonen K. Is exposure to poultry harmful to child nutrition? An observational analysis for rural Ethiopia. *PLoS One*. 2016;11(8):1–16.

doi:https://doi.org/10.1371/journal.pone.0160590

55. Ngure F, Gelli A, Becquey E, et al. Exposure to livestock feces and water quality, sanitation, and hygiene (wash) conditions among caregivers and young children: Formative research in rural Burkina Faso. *Am J Trop Med Hyg*. 2019;100(4):998-1004. doi:10.4269/ajtmh.18-0333

56. Exum NG, Olórtegui MP, Yori PP, et al. Floors and Toilets: Association of Floors and Sanitation Practices with Fecal Contamination in Peruvian Amazon Peri-Urban Households. *Environ Sci Technol*. 2016;50:7373−7381. doi:10.1021/acs.est.6b01283

57. George C, Oldja L, Biswas S et al. Geophagy is associated with environmental enteropathy and stunting in children in rural Bangladesh. *Am J Trop Med Hyg*. 2015;92(6):1117–1124. doi:10.4269/ajtmh.14-0672

58. Pickering A, Julian T, Marks S et al. Fecal contamination and diarrheal pathogens on surfaces and in soils among Tanzanian households with and without improved sanitation. *Envir Sci Technol*. 2012;46(11):5736–5743. doi:10.1021/es300022c

59. Central Statistical Agency-CSA/Ethiopia I. *Ethiopia Demographic and Health Survey (DHS) 2016*. Addis Ababa, Ethiopia; 2017. doi:10.2147/ahmt.s148434

60. Sebastian K. *Atlas of African Agriculture Research & Development: Revealing Agriculture's Place in Africa*. Washington DC: International Food Policy Research Institute (IFPRI); 2014. doi:http://dx.doi.org/10.2499/9780896298460

61. Save the Children, The Manoff Group Inc. *Water, Hygiene and Sanitation (WASH) in Rural Households in Amhara, Oromia, SNNP and Tigray. A Report on the Findings and Recommendations of a Multi-Method Qualitative Research Study for Social and Behavior Change Communication Programming in Ethiopia.* Addis Ababa, Ethiopia; 2014.

62. Gedlu E, Aseffa A. Campylobacter enteritis among children in north-west Ethiopia: A 1-year prospective study. *Ann Trop Paediatr*. 1996;16(3):207-212. doi:10.1080/02724936.1996.11747828

63. Mitike G, Kassu A, Genetu A, Nigussie D. Campylobacter enteritis among children in

Dembia District, Northwest Ethiopia. *East Afr Med J*. 2009;77(12):654-657. doi:10.4314/eamj.v77i12.46764

64. Mulatu G, Beyene G, Zeynudin A. Prevalence of Shigella, Salmonella and Campylobacter species and their susceptibility patters among under five children with diarrhea in Hawassa town, south Ethiopia. *Ethiop J Health Sci*. 2014;24(2):101-108.

65. Gittelsohn J, Steckler A, Johnson CC, et al. Formative research in school and community-based health programs and studies: "State of the art" and the TAAG approach. *Heal Educ Behav*. 2006. doi:10.1177/1090198105282412

66. Baqui AH, Black RE, Yunus M, Hoque ARA, Chowdhury HR, Sack RB. Methodological issues in diarrhoeal diseases epidemiology: Definition of diarrhoeal episodes. *Int J Epidemiol*. 1991. doi:10.1093/ije/20.4.1057

67. de Onis M, Onyango AW, Van den Broeck J, Chumlea WC, Martorell R. Measurement and standardization protocols for anthropometry used in the construction of a new international growth reference. *Food Nutr Bull*. 2004;25(1):S27-S36.

68. CHROMagar. CHROMagarTM Campylobacter: Instructions for use. Version 3.0.

69. Bensersa-Nedjar D, Zerouki A, Aggoune N, Yamouni F, Henniche F-Z, Chabani A. *Evaluation Comparative de Trois Milieux de Culture Sélectifs ; CHROMagar Campylobacter (CHROMagar), Karmali (Oxoid) et Campylosel (BioMérieux), Pour La Recherche Des Campylobacter Thermotolérants à Partir Des Échantillons Fécaux*. Alger: Algérie; 2017.

70. Forsberg L, Barth C, Malejczyk K, Minion J. *Evaluation of CHROMagar Campylobacter (CAC)*. Regina, SK. S4P 0W5; 2017.

71. De Zutter L, Van Damme I, Mugg P, Ukaegbu K. *Comparison of Four Different Selective Media for the Quantification of Campylobacter in Poultry Meat and Rapid Confirmation of Suspect Colonies*. Ghent; 2017.

72. WHO Multicentre Growth Reference Study Group. *WHO Child Growth Standards. Length/Height-for-Age, Weight-for-Age, Weight-for-Length, Weight-for-Height and*

Body Mass Index-for-Age: Methods and Development. Department of Nutrition Health and Development. Geneva: World Health Organization; 2006.

73. Rogawski McQuade ET, Platts-Mills JA, Gratz J, et al. Impact of Water Quality, Sanitation, Handwashing, and Nutritional Interventions on Enteric Infections in Rural Zimbabwe: The Sanitation Hygiene Infant Nutrition Efficacy (SHINE) Trial. *J Infect Dis*. 2019;jiz179. doi:10.1093/infdis/jiz179

74. Randremanana R V., Randrianirina F, Sabatier P, et al. Campylobacter infection in a cohort of rural children in Moramanga, Madagascar. *BMC Infect Dis*. 2014;14(372):1-9. doi:10.1186/1471-2334-14-372

75. Mason J, Iturriza-Gomara M, O'Brien SJ, et al. Campylobacter Infection in Children in Malawi Is Common and Is Frequently Associated with Enteric Virus Co-Infections. *PLoS One*. 2013;8(3):e59663. doi:10.1371/journal.pone.0059663

76. MAL-ED Network Investigators. Relationship between growth and illness, entereopathogens and dietary intakes in the first 2 years of life. *BMJ Glob Heal*. 2017;2(e000370). doi:10.1136/ bmjgh-2017-000370

77. Headey D, Nguyen P, Kim S, Rawat R, Ruel M, Menon P. Is exposure to animal feces harmful to child nutrition and health outcomes? A multicountry observational analysis. *Am J Trop Med Hyg*. 2017;96(4):961–969.

78. Schoenbuchner SM, Dolan C, Mwangome M, et al. The relationship between wasting and stunting: a retrospective cohort analysis of longitudinal data in Gambian children from 1976 to 2016. *Orig Res Commun Am J Clin Nutr*. 2019;109:1-10. doi:10.1093/ajcn/nqy326.

79. Martorell R, Young MF. Patterns of Stunting and Wasting: Potential Explanatory Factors. *Adv Nutr*. 2012;3(2):227-233. doi:10.3945/an.111.001107

80. Reid B, Orgle J, Roy K, Pongolani C, Chileshe M, Stoltzfus R. Characterizing potential risks of fecal–oral microbial transmission for infants and young children in Rural Zambia. *Am J Trop Med Hyg*. 2018. doi:10.4269/ajtmh.17-0124

81. Yeager B, Lanata C, Lazo F, Verastegui H, Black R. Transmission factors and

socioeconomic status as determinants of diarrhoeal incidence in Lima, Peru. *J Diarrhoeal Dis Res*. 1991;9(3):186–193. doi:https://www.jstor.org/stable/23498080

82. Lengerh A, Moges F, Unakal C, Anagaw B. Prevalence, associated risk factors and antimicrobial susceptibility pattern of Campylobacter species among under five diarrheic children at Gondar University Hospital, Northwest Ethiopia. *BMC Pediatr*. 2013;13(82):1-9. doi:10.1186/1471-2431-13-82

83. Tafa B, Sewunet T, Tassew H, Asrat D. Isolation and Antimicrobial Susceptibility Patterns of Campylobacter Species among Diarrheic Children at Jimma, Ethiopia. *Int J Bacteriol*. 2014;2014(560617). doi:10.1155/2014/560617

84. Wagner E, Lanoix J. *Excreta Disposal for Rural Areas and Small Communities.* Vol 39.; 1958.

85. Schnee AE, Haque R, Taniuchi M, et al. Identification of Etiology-Specific Diarrhea Associated with Linear Growth Faltering in Bangladeshi Infants. *Am J Epidemiol*. 2018;187(10):2210–2218. doi:10.1093/aje/kwy106

86. Buss JE, Cresse M, Doyle S, Buchan BW, Craft DW, Young S. Campylobacter culture fails to correctly detect Campylobacter in 30% of positive patient stool specimens compared to non-cultural methods. *Eur J Clin Microbiol Infect Dis*. 2019;38(6):1087–1093. doi:10.1007/s10096-019-03499-x

87. do Nascimento Veras H, da Silva Quetz J, Lima IFN, et al. Combination of different methods for detection of Campylobacter spp. in young children with moderate to severe diarrhea. *J Microbiol Methods*. 2016;128:7-9. doi:10.1016/j.mimet.2016.06.026

88. Lin A, Arnold B, Afreen S et al. Household environmental conditions are associated with enteropathy and impaired growth in rural bangladesh. *Am J Trop Med Hyg*. 2013;89(1):130–137. doi:10.4269/ajtmh.12-0629

89. Pickering A, Null C, Winch P, Mangwadu G, Arnold B, Prendergast A. The WASH Benefits and SHINE trials: interpretation of WASH intervention effects on linear growth and diarrhoea. 2019;7(8):E1139-E1146. doi:10.1016/S2214-109X(19)30268-2

90. Husseini M, Darboe MK, Moore SE, Nabwera HM, Prentice AM. Thresholds of socio-

economic and environmental conditions necessary to escape from childhood malnutrition: A natural experiment in rural Gambia. *BMC Med*. 2018;16(1):199. doi:10.1186/s12916-018-1179-3

Reviewer	Comment	Change made (SB)
Editor	Table 1, I would suggest change the header “% of total” to “Average or Percent”, and move the values of “average age” and “average duration of diarrhea” from the “n” column to the “Average or Percent” column. Please also give the counts of diarrhea during last 7 days.	We thank the editor for their personal feedback on the manuscript and believe the comments have helped improve the overall draft. We appreciate the opportunity to respond to these comments.<br><br>This change has been made in Table 1. I believe the count of diarrhoea during the last 7 days is already noted (n=11) – apologies if we have misinterpreted your suggestion.
	Page 13, lines 316-318. This sentence is very unclear to me. Are you comparing prevalence of infection between children with diarrhea during past 7 days and those without? If so, there should be an odds ratio. How are “symptomatic” vs “asymptomatic” carriers defined? Infections with diarrhea and infections without diarrhea in past 7 days?	Thank you. Yes, symptomatic and asymptomatic are defined as you mention (with and without diarrhoea respectively, where diarrhoea was current [the stool sample was diarrhoeal]). This is clarified in lines 291-293 and again lines 335-336.
	Line 321, how was the OR of 1.41 calculated? If wasted kids were all infected, the odds of infection among wasted kids is then infinity, and the OR would be infinity too, as long as the infection rate among non-wasted children is not 100%. Given 95% CI for these ratios as well.	Thank you. This has been worded wrongly as all infants were compared. This is amended. This has also been amended in lines 338-343.
	I would suggest making a table for all important OR estimates.	This is included, thank you.
	Figure 1, again, not sure what is the difference between “symptomatic (diarrhea)” and “Diarrhea within past 7 days”. One is 58.8% and the other is 31.4%, so they have to be different. Please give clear definition.	Thank you. We classified ‘symptomatic’ as infants who tested positive for Campylobacter who also had a diarrhoeal stool. Diarrhoea during the last 7 days was not tested for an association with positive infant faeces (although one might argue that it should be). This has been clarified again in lines 291-293 and lines 335-336. We have also added some detail in the figure legend (Fig 1, lines 372-373) if this helps to clarify, but can be removed if more appropriate.
	Were poultry feces collected indoor or outdoor? Were poultry feces common indoor?	This is clarified lines 221-222. Thank you for making this point also, it is valuable: we did note the samples that came from outdoors versus indoors and I have made this point in the discussion (lines 425-427).
	If you developed a questionnaire as part of this study and it is not under a copyright more restrictive than CC-BY, please include a	This is included, thank you.

	copy, in both the original language and English, as Supporting Information.	
	Funding information should not appear in the Acknowledgments section or other areas of your manuscript.	This is removed.
**1**	Without confirmation of the taxonomic nature of the isolates by molecular and sequencing approach, I am highly suspecting that the Campylobacter prevalence reported in this study are highly overestimated. Further recovering Campylobacter from field samples can be very challenging in low income countries (I speak from experience); therefore, the use of a direct plating approach might not be the best strategy. This approach must be combined with more sensitive and specific methods.	The authors thank the reviewer for their valuable comments on our paper and we welcome the opportunity to change the manuscript to align with this feedback.<br><br>We would like to present several studies which have used culture-based methods alone to detect Campylobacter in low-income countries, including:<br>1. El-Tras WF et al. *Epidemiol. Infect.* (2015), 143: 308–315<br>2. Grados O et al. *Bulletin of the World Health Organization* (1988), 66 (3): 369-374<br>3. Lee G et al. *PLoS Negl Trop Dis.* (2013), 7(1): e2036<br>4. Rao MR et al. *Am J Epidemiol.* (2001), 154(2): 166-173<br>And also in Ethiopia:<br>5. Kassie GM et al. *East Afr Med J.* (2000) 77(12): 654-657<br>6. Gedlu E and Aseffa A. *Ann Trop Paediatr.* (1996), 16(3): 207-212<br>We respectfully disagree with the reviewer that culture overestimates Campylobacter infection and may actually underestimate due to VBNC (viable but non-culturable) bacteria. This is already noted in the limitations in lines 495-504. The authors are aware that there will be a rate of false positives that we cannot determine, and we have added this to the limitations section (line 501).<br>Whilst PCR demonstrates high sensitivity, there are also pitfalls to using this detection method. Indeed it has also been noted that PCR will pick up Campylobacter of low burden, potentially over-estimating infection prevalence. Further, qPCR can also overestimate viable

		cells due to the presence of extracellular DNA which contributes to the signal.<br>Culture alone is not a perfect method but in this study, was used as a simple method to provide formative data towards a hypothesis.<br>We hope this response addresses the reviewer's important concern.
	**Abstract:**	
	Missing word in the sentence line 25-26	Thank you. We cannot identify a missing word here and think the sentence reads clearly.
	All numbers starting at the beginning of a sentence must be fully written (not numeric)	Thank you, this has been changed throughout.
	Full name of SNNPR	This has been changed.
	Please provide the r2 for the correlation data between the campylobacter prevalence and other variables studied (line 42)	The authors respectfully comment that it is possible the reviewer has misinterpreted the analysis performed in this paper. As stated in the statistical analysis section (lines 301-304), we used Fisher's exact test to test associations between variables which predicted positive infant faeces. We used Fisher's exact test as a more appropriate measure for correlation due to the small sample size, which also gives an odds ratio and corresponding p-value, as stated in the text.<br>We did not perform a regression between variables as it was felt by the team that the sample size was not sufficiently large to perform such an analysis and we felt we would be 'over-analysing' the data. As such, a limitation is that the associations here are not part of a model where other factors would be held constant/controlled for, but present associations between exposure variables and the outcome.
	Only the use of microbiology approach (direct plating on selective medium) to identify Campylobacter is not good sufficient enough. Additional molecular approach (PCR) must be performed to confirm the isolates belong to the Campylobacter genus.	We thank you reviewer for their comment and insight here. We have addressed this particular comment above.
	**Introduction:**	
	The introduction is too long	We thank the reviewer for their comment on the introduction. We politely disagree that the

Commented [BS1]: Here

		introduction is longer than is usually submitted to PLOS One. We endeavoured to frame the study within current evidence and to address the research need. We will be happy to cut the introduction if further reviews suggest to.
	**Materials and Methods**	
	Remove text from line 161 to 167.	We thank the reviewer for this suggestion. We respectfully choose to keep the sentence to frame the study within context. The second reviewer also suggests to include more context-specific examples of research within the field.
	Line 173: Replace Zone by zone. Further, what do you mean by zone (=Woreda or kebele)?	Thank you, this is clarified in line 176.
	Line 173-175, More details are required concerning the simple random sampling method. Did the number of free-range chickens per household was recorded? Did the authors saw a correlation between the campylobacter prevalence or other recorded data and the number of free-range chickens.	Thank you. The random sample is already described in the text following (lines 180-188). A line has been added to clarify that the description follows.
	Line 178, “simple lottery method” Can you clarify?	Thank you. The simple lottery method is clarified within the description.
	Line 180-182: I strongly disagree with the authors. The authors used these data to estimate the prevalence of Campylobacter in this specific area in Ethiopia. Therefore, the sampling method and the sample size are crucial in order to properly estimate the prevalence of Campylobacter. Please remove this sentence. Further, the authors must acknowledge that the population size low and restricted to only two kebeles. Please can you provide information concerning the expected statistical power of your analysis based population size. These information must be acknowledge in the manuscript as limitations of your study	We thank the reviewer for their feedback here. We respect the view, however we politely disagree here. Formative research is not intended to test a hypothesis, but is intended as an initial part of the process of a larger study design, and can use both qualitative and quantitative methods to provide data for research teams to plan interventions or further data collection. Formative data is early phase data not powered to detect differences between groups (Gittelsohn et al., 2006).<br>We have added this description to the methods (lines 190-196) to clarify the positioning of this study. We hope this addresses the reviewer’s concern.
	Line 194-195, please can you explain how the comparison was performed. Did you have reference chart for the consistency (i.e. solid, pasty, liquid)?	Thank you for your comment. The reporting of caregiver-reported diarrhoea is a standard measurement using WHO guidelines, as is referenced in lines 203-206. We have also described in lines 206-208 how we matched with visible diarrhoea. We did not use a chart

		but diarrhoea was clear from samples as is stated (6 or 7 on the Bristol stool chart).
	Line 202: what is the estimated time between the sampling and the incubation of the inoculated selective plates in microaerophilic conditions?	Thank you, we assume this refers to line 227 and have added a sentence to clarify timing.
	Line 220: did the authors used a positive control in parallel during the processing the field samples (i.e. pure culture of C. jejuni or C. coli for example)?	Thank you. We did not use a positive control within the field. As described in lines 263-266, the media was tested prior to fieldwork using a pure culture and blank samples were used in the field to confirm absence of contamination.
	Line 233: For how long the plates drying? This step is not necessary and might cause the death of Campylobacter.	Thank you for your insight. We left the plates to dry as per manufacturer instructions. (CHROMagar) before stacking the plates. Indeed other teams reporting similar work in PLOS NTDs stated they left the inoculation to passively diffuse for 30 minutes (Lee G et al. PLoS Negl Trop Dis. (2013), 7(1): e2036).<br>We left the sample to dry for approximately 5 minutes. This is added in line 252-253.
	Line 233-237: I know by experience that this protocol is not sufficient to isolate Campylobacter from feces in Ethiopia due to the development of dense background (green colonies for example) and/or the presence of red campylobacter-like colonies that are not Campylobacter after confirmation via genus specific PCR (16S primer).	We thank the reviewer for their insight. We are unsure how this sentence aligns with lines 233-237 in the manuscript.<br>We note in lines 261-263 the prior testing of this Chromagar which shows high sensitivity and specificity. We did see a dense background in some plates when incubated: However, we discussed this with the manufacturer. It was stated that Chromagar media is designed for the isolation of thermotolerant Campylobacter (jejuni, lari, coli) and is very good at suppressing growth of background flora (the enterics such as E.coli and Enterococcus); thus there is confidence that any red colonies on the plate are Campylobacter spp.<br><br>Without confirming these results with PCR or other tests, there is little way we can respond: however, we trust in the validity of findings which have shown Chromagar selects for Campylobacter, and have indicated that Chromagar was used for the isolation and

		**presumptive identification** of thermotolerant Campylobacter (line 240).<br>To avoid over-claiming on our findings, we use the term 'presumptive' where possible in the manuscript.<br><br>We would also like to respectfully express we think it is important that these sorts of studies are replicated across different settings and contexts. Where the media may not have been sufficient in one context, that may not be the case across all studies. As with any scientific area, it is important to have more than one set of findings to make a judgement: particularly with new methods (Chromagar) where other authors may replicate protocols and add further evidence.
	Line 241-243: Unfortunately, this statement is not true. Our studies (unpublished data) showed that other genus can grow in this medium, which give campylobacter-like colonies. Due date, the only way we could differentiate them was via aerobic growth test or 16S sequencing.	Thank you for your comment and sharing your experience in the field. We believe we have addressed this comment above.<br>We would like to stress the same point made above about the importance of sharing findings from similar methodologies in order to strengthen the area of research.
	Line 249-252: I am scared that without PCR confirmation, you cannot affirm that your isolates are from the Campylobacter genus. This limitation must be acknowledged in your manuscript. I am suspecting that you are highly overestimating the Campylobacter prevalence based on your direct plating data. Our team faced the exact same issues in a previous project. Out of 1200 suspected Campylobacter isolates collected in Ethiopia using CHROMagar, less them 5% of the isolates were positive for Campylobacter via PCR. I would recommend buying external generators, which allow to continue your PCR cycles for 3-6 hrs.	We thank the reviewer for this insight and for sharing their experience. We believe we have addressed this comment with our first comment in response to the same comment from them.<br>We agree an external generator would have been great – indeed back-up generators also failed on this occasion.
	Line 273: you do not know whether Campylobacter or another microorganism of the gut microbiota is the causal agent of the diarrhea symptoms observed in the infant; thereby, you cannot use the term infected. It needs to be changed throughout the manuscript.	Apologies we cannot see how this comment refers to line 273.<br>If the reviewer refers to lines 388 onwards, we have described that this is an association and the relationship with the excretion period of Campylobacter within the stool. We add a line that of course other microorganisms may be

		present which caused diarrhoeal symptoms (lines 397-398).<br><br>Respectfully, we would like to add the point that if we see suspected growth in a stool sample, it is highly likely the infant is infected – Campylobacter is not normally isolated in a stool, and only very small amount of subtypes are part of 'commensal' gut flora – most of them are disease-causing.
	**Results:**	
	Line 311-323: You cannot use the term infected. It needs to be changed throughout the manuscript. All correlation data presented in the manuscript should be associated with a r2 value to fully appreciated the power of the correlation.	Thank you for your comment. As described in the point above, we have described the association. We acknowledge in our paper that Campylobacter is likely not the only cause of infection. We have also altered how we determine 'infection' in this paper, as infant stools 'positive or negative' for presumptive Campylobacter (lines 288-293). We do not wish to appear we are over-claiming on the data and as such, we have edited both the long and short titles. We hope these are appropriate and reflect the limitations of the small sample size.<br><br>We thank the reviewer for their suggestion on the r2 value; we have responded to this in a comment above.
	Line 311: Did you observed difference in Campylobacter prevalence between the two kebeles. Why the authors did not present the CFU data from the direct plating?	Thank you for your comment and questions. We did not separate between the two kebeles. Given the small sample size, we did not believe this would give a meaningful finding. We did not present CFU as this was not the primary aim of the study, we intended to gauge prevalence only.
	Line 341-342: This sentence is for the discussion, not the results	Apologies but we cannot see how lines 341-342 is intended for discussion.
	Line 343-34: This sentence is for the fig legend, not the results	Again apologies but we cannot see how these lines should be removed. We have removed two lines, 360 and 364 if these are those that the reviewer referred to.

Commented [BS2]: Here

	Line 346: the legend in fig 1 and 2 should explain what the p values means. Further the quality of the figures is not good enough (too pixeled).	Thank you for your suggestion. We are unsure exactly what the reviewer means by 'explain what p value means' but have added the statement as to the significance boundary to Figure 1. Figure 2 does not contain any p values.<br>Upon submission to PLOS One the figure file types will be of sufficient quality as requested.
	Line 364: the author must acknowledge the other potential reason of the diarrhea (other microorganism of the microbiota)	Thank you. We think this request refers to the wrong line in the text. We have added this statement in lines 398-399.
	Line 366-368: the population is too small, you cannot do such hypothesis.	Again apologies but we do not think the line the reviewer is referring to corresponds to the manuscript. We have included caveats throughout the manuscript as to limitations with the size of the sample.
	Line 375-377: your data do not support that given you don't know if campylobacter is the causal agent of the diarrhea.	Thank you. Again we have amended lines 398-399 and is already acknowledged in lines 491-494.
	Line 398-400: Again your population size is too small to make any hypothesis or conclusion. You may suggest a potential transmission from floor to infant but more studies must be done to confirm these preliminary results!	Again we are unsure which line the reviewer refers to. On line 435, we have added 'potentially'. However many other studies have demonstrated the importance of contaminated floors as an important transmission pathway to infants due to faeces and contamination of the floor surface (including references 27,49,50 on line 137, references 56-58 on line 154 and references 27, 80, 81 on line 445).
	Line 417-419; 453-455; 473-476: same comment then above. Your population size is too small to make any hypothesis or conclusion. These are preliminary results.	Again we are unsure which line the reviewer refers to. In line 481-484 we have stressed the importance that the results are formative and the sample is small.
	The discussion is long and the authors miss to mention anything about the important limitation of this study: the population size and the lack of complete methodology to confirm the nature of the isolates.	Thank you for your comment. We agree the discussion is long; we also included the conclusion. We have removed some paragraphs to reduce the overall length.<br>We add caveats and limitations throughout the discussion, including:<br>Lines 154-155<br>Line 175<br>Lines 190-196<br>Lines 481-484

		Lines 496-505
**2**	**Abstract:**	
	Add specific stud area: SNNO has over 14,000,000 population. the sidama population is over 6 million	We thank the reviewer for their comments on the manuscript and we are pleased to send our responses.<br><br>Thank you, we have added Sidama zone.
	Add keyword at the end.	Thank you, we have added keywords after the abstract.
	Any association between improved wash and children growth.	Thank you, we think this is already addressed in lines 45-46.
	I do not think there is a author summery after abstract. If not remove it.	Thank you, PLOS One indeed does not require an author summary, this is removed.
	**Introduction:**	
	Add local studies on prevalence of wash, campylobacter, and stunting in Ethiopia.	Thank you for your suggestion. We have added two studies (line 169-172) and also a further study which was conducted in Hawassa city in the same zone. We have referenced these again in the discussion (lines 393-394).
	**Methods:**	
	Add detail methods of standardization and cite them	Thank you. We have noted some use of standardized methods in the methodology section. The survey was previously validated (line 199). The Chromagar is used and prepared according to manufacturer's instructions (line 252). Anthropometry is performed according to WHO guidelines (reference added, line 213).
	Is anthropometry is recommended for children 0-6 months, justify it or include in limitation of the study	Thank you. This study does not include infants under the age of 10 months, for whom anthropometry is appropriate.
	Cite the acceptability of your sample collection and transport methods in low income setting.	Thank you for the suggestion. We have cited some similar research within Ethiopia which used collection and transport methods similar to our study (line 231).
	**Results and discussion:**	
	I did not found the regression table in the section.	Thank you. As described in the statistical analysis section (lines 301-304), we used the Fisher's exact test to test associations between variables. Fisher's exact test is a more

		appropriate measure for correlation in this study due to the small sample size. The test also gives an odds ratio and corresponding p-value, as stated in the text.<br>We did not perform a regression between variables as it was felt by the team that the sample size was not large enough.
	Add strength and limitation of this study	Thank you for your comment. As we replied to the first reviewer, we have included caveats and limitations throughout the paper including points about formative research (lines 190-196) and refer to limitations within the discussion, including lines 481-505.

Commented [BS3]: Here
