# Supplementary material for: Risk factors and transmission pathways associated with infant Campylobacter spp. prevalence and malnutrition: A formative study in rural Ethiopia
Source: PLoS One. 2020 May 8;15(5):e0232541. doi: 10.1371/journal.pone.0232541 (PMC7209302; doi:10.1371/journal.pone.0232541)
Supplement: Attachment — Submitted filename: review.docx [file pone.0232541.s002.docx]

The results described in this study represent a hot topic associated with important public health issues that need to be remediated as soon as possible. The manuscript was well written and I really appreciate the clarity and creativity of the figures, which really help in the interpretation of the data. Overall the study have great potential to help national and international institutes to fight *Campylobacter* transmission to infant and EED in Ethiopia; however major pitfalls are remaining in this study. For example, the suspected *Campylobacter* isolates isolated on CHROMagar were not confirmed via PCR due to technical difficulties and the population samples is low (n=35 infants). Without confirmation of the taxonomic nature of the isolates by molecular and sequencing approach, I am highly suspecting that the Campylobacter prevalence reported in this study are highly overestimated. Further recovering Campylobacter from field samples can be very challenging in low income countries (I speak from experience); therefore, the use of a direct plating approach might not be the best strategy. This approach must be combined with more sensitive and specific methods.

**Abstract**

- Missing word in the sentence line 25-26
- All numbers starting at the beginning of a sentence must be fully written (not numeric)
- Full name of SNNPR
- Please provide the r2 for the correlation data between the campylobacter prevalence and other variables studied (line 42)
- Only the use of microbiology approach (direct plating on selective medium) to identify Campylobacter is not good sufficient enough. Additional molecular approach (PCR) must be performed to confirm the isolates belong to the *Campylobacter* genus.

**Introduction**

- The introduction is too long

**Materials and Methods**

- Remove text from line 161 to 167.
- Line 173: Replace Zone by zone. Further, what do you mean by zone (=Woreda or kebele)?
- Line 173-175, More details are required concerning the simple random sampling method. Did the number of free-range chickens per household was recorded? Did the authors saw a correlation between the campylobacter prevalence or other recorded data and the number of free-range chickens.
- Line 178, “simple lottery method” Can you clarify?
- Line 180-182: I strongly disagree with the authors. The authors used these data to estimate the prevalence of *Campylobacter* in this specific area in Ethiopia. Therefore, the sampling method and the sample size are crucial in order to properly estimate the prevalence of *Campylobacter*. Please remove this sentence. Further, the authors must acknowledge that the population size low and restricted to only two kebeles. Please can you provide information concerning the expected statistical power of your analysis based population size. These information must be acknowledge in the manuscript as limitations of your study.
- Line 194-195, please can you explain how the comparison was performed. Did you have reference chart for the consistency (i.e. solid, pasty, liquid)?
- Line 202: what is the estimated time between the sampling and the incubation of the inoculated selective plates in microaerophilic conditions?
- Line 220: did the authors used a positive control in parallel during the processing the field samples (i.e. pure culture of C. jejuni or C. coli for example)?
- All numbers starting at the beginning of a sentence must be fully written (not numeric)
- Line 233: For how long the plates drying? This step is not necessary and might cause the death of *Campylobacter.*
- Line 233-237: I know by experience that this protocol is not sufficient to isolate Campylobacter from feces in Ethiopia due to the development of dense background (green colonies for example) and/or the presence of red campylobacter-like colonies that are not Campylobacter after confirmation via genus specific PCR (16S primer).
- Line 241-243: Unfortunately, this statement is not true. Our studies (unpublished data) showed that other genus can grow in this medium, which give campylobacter-like colonies. Due date, the only way we could differentiate them was via aerobic growth test or 16S sequencing.
- Line 249-252: I am scared that without PCR confirmation, you cannot affirm that your isolates are from the *Campylobacter* genus. This limitation must be acknowledged in your manuscript. I am suspecting that you are highly overestimating the *Campylobacter* prevalence based on your direct plating data. Our team faced the exact same issues in a previous project. Out of 1200 suspected *Campylobacter* isolates collected in Ethiopia using CHROMagar, less them 5% of the isolates were positive for *Campylobacter* via PCR. I would recommend buying external generators, which allow to continue your PCR cycles for 3-6 hrs.
- Line 273: you do not know whether *Campylobacter* or another microorganism of the gut microbiota is the causal agent of the diarrhea symptoms observed in the infant; thereby, you cannot use the term infected. It needs to be changed throughout the manuscript.

**Results**

- Line 311-323: You cannot use the term infected. It needs to be changed throughout the manuscript. All correlation data presented in the manuscript should be associated with a r2 value to fully appreciated the power of the correlation.
- Line 311: Did you observed difference in *Campylobacter* prevalence between the two kebeles. Why the authors did not present the CFU data from the direct plating?
- Line 341-342: This sentence is for the discussion, not the results
- Line 343-34: This sentence is for the fig legend, not the results
- Line 346: the legend in fig 1 and 2 should explain what the p values means. Further the quality of the figures is not good enough (too pixeled).

**Discussion**

- Line 364: the author must acknowledge the other potential reason of the diarrhea (other microorganism of the microbiota)
- Line 366-368: the population is too small, you cannot do such hypothesis.
- Line 375-377: your data do not support that given you don’t know if campylobacter is the causal agent of the diarrhea.
- Line 398-400: Again your population size is too small to make any hypothesis or conclusion. You may suggest a potential transmission from floor to infant but more studies must be done to confirm these preliminary results!
- Line 417-419; 453-455; 473-476: same comment then above. Your population size is too small to make any hypothesis or conclusion. These are preliminary results.
- The discussion is long and the authors miss to mention anything about the important limitation of this study: the population size and the lack of complete methodology to confirm the nature of the isolates.
