# Supplementary material for: Risk factors and transmission pathways associated with infant Campylobacter spp. prevalence and malnutrition: A formative study in rural Ethiopia
Source: PLoS One. 2020 May 8;15(5):e0232541. doi: 10.1371/journal.pone.0232541 (PMC7209302; doi:10.1371/journal.pone.0232541)
Supplement: Attachment — Submitted filename: plos reveiw response.docx [file pone.0232541.s003.docx]

Abstract:

Add specific stud area: SNNO has over 14,000,000 population. the sidama population is over 6 million

Add keyword at the end.

Any association between improved wash and children growth.

I donot think there is a author summery after abstract . If not remove it.

Introduction

Add local studies on prevalence of wash, campylobacter, and stunting in Ethiopia.

Methods:

Add detail methods of standardization and cite them

Is anthropometry is recommended for children 0-6 months, justify it or include in limitation of the study

Cite the acceptability of your sample collection and transport methods in low income setting.

Is all process of isolation of campylobacter spp. Is done/guided by single publication(61).

Results and discussion:

I did not found the regression table in the section.

Add strength and limitation of this study
