# Supplementary material for: Risk factors and transmission pathways associated with infant Campylobacter spp. prevalence and malnutrition: A formative study in rural Ethiopia
Source: PLoS One. 2020 May 8;15(5):e0232541. doi: 10.1371/journal.pone.0232541 (PMC7209302; doi:10.1371/journal.pone.0232541)
Supplement: Attachment — Submitted filename: Reviewer responses_060220.docx [file pone.0232541.s004.docx]

| **Reviewer** | **Comment** | **Change made (SB)** |
| --- | --- | --- |
| **Editor** | Table 1, I would suggest change the header “% of total” to “Average or Percent”, and move the values of “average age” and “average duration of diarrhea” from the “n” column to the “Average or Percent” column. Please also give the counts of diarrhea during last 7 days. | We thank the editor for their personal feedback on the manuscript and believe the comments have helped improve the overall draft. We appreciate the opportunity to respond to these comments.  This change has been made in Table 1. I believe the count of diarrhoea during the last 7 days is already noted (n=11) – apologies if we have misinterpreted your suggestion. |
|  | Page 13, lines 316-318. This sentence is very unclear to me. Are you comparing prevalence of infection between children with diarrhea during past 7 days and those without? If so, there should be an odds ratio. How are “symptomatic” vs “asymptomatic” carriers defined? Infections with diarrhea and infections without diarrhea in past 7 days? | Thank you. Yes, symptomatic and asymptomatic are defined as you mention (with and without diarrhoea respectively, where diarrhoea was current [the stool sample was diarrhoeal]). This is clarified in lines 291-293 and again lines 335-336. |
|  | Line 321, how was the OR of 1.41 calculated? If wasted kids were all infected, the odds of infection among wasted kids is then infinity, and the OR would be infinity too, as long as the infection rate among non-wasted children is not 100%. Given 95% CI for these ratios as well. | Thank you. This has been worded wrongly as all infants were compared. This is amended. This has also been amended in lines 338-343. |
|  | I would suggest making a table for all important OR estimates. | This is included, thank you. |
|  | Figure 1, again, not sure what is the difference between “symptomatic (diarrhea)” and “Diarrhea within past 7 days”. One is 58.8% and the other is 31.4%, so they have to be different. Please give clear definition. | Thank you. We classified ‘symptomatic’ as infants who tested positive for Campylobacter who also had a diarrhoeal stool. Diarrhoea during the last 7 days was not tested for an association with positive infant faeces (although one might argue that it should be). This has been clarified again in lines 291-293 and lines 335-336. We have also added some detail in the figure legend (Fig 1, lines 372-373) if this helps to clarify, but can be removed if more appropriate. |
|  | Were poultry feces collected indoor or outdoor? Were poultry feces common indoor? | This is clarified lines 221-222. Thank you for making this point also, it is valuable: we did note the samples that came from outdoors versus indoors and I have made this point in the discussion (lines 425-427). |
|  | If you developed a questionnaire as part of this study and it is not under a copyright more restrictive than CC-BY, please include a copy, in both the original language and English, as Supporting Information. | This is included, thank you. |
|  | Funding information should not appear in the Acknowledgments section or other areas of your manuscript. | This is removed. |
| **1** | Without confirmation of the taxonomic nature of the isolates by molecular and sequencing approach, I am highly suspecting that the Campylobacter prevalence reported in this study are highly overestimated. Further recovering Campylobacter from field samples can be very challenging in low income countries (I speak from experience); therefore, the use of a direct plating approach might not be the best strategy. This approach must be combined with more sensitive and specific methods. | The authors thank the reviewer for their valuable comments on our paper and we welcome the opportunity to change the manuscript to align with this feedback.  We would like to present several studies which have used culture-based methods alone to detect Campylobacter in low-income countries, including:   1. El-Tras WF et al. *Epidemiol. Infect.* (2015), 143: 308–315 2. Grados O et al. *Bulletin of the World Health Organization* (1988)*,* 66 (3): 369-374 3. Lee G et al. *PLoS Negl Trop Dis.* (2013), 7(1): e2036 4. Rao MR et al. *Am J Epidemiol.* (2001), 154(2): 166-173   And also in Ethiopia:   5. Kassie GM et al. *East Afr Med J.* (2000) 77(12): 654-657 6. Gedlu E and Aseffa A. *Ann Trop Paediatr.* (1996), 16(3): 207-212   We respectfully disagree with the reviewer that culture overestimates Campylobacter infection and may actually underestimate due to VBNC (viable but non-culturable) bacteria. This is already noted in the limitations in lines 495-504. The authors are aware that there will be a rate of false positives that we cannot determine, and we have added this to the limitations section (line 501).  Whilst PCR demonstrates high sensitivity, there are also pitfalls to using this detection method. Indeed it has also been noted that PCR will pick up Campylobacter of low burden, potentially over-estimating infection prevalence. Further, qPCR can also overestimate viable cells due to the presence of extracellular DNA which contributes to the signal.  Culture alone is not a perfect method but in this study, was used as a simple method to provide formative data towards a hypothesis.  We hope this response addresses the reviewer’s important concern. |
|  | **Abstract:** |  |
|  | Missing word in the sentence line 25-26 | Thank you. We cannot identify a missing word here and think the sentence reads clearly. |
|  | All numbers starting at the beginning of a sentence must be fully written (not numeric) | Thank you, this has been changed throughout. |
|  | Full name of SNNPR | This has been changed. |
|  | Please provide the r2 for the correlation data between the campylobacter prevalence and other variables studied (line 42) | The authors respectfully comment that it is possible the reviewer has misinterpreted the analysis performed in this paper. As stated in the statistical analysis section (lines 301-304), we used Fisher’s exact test to test associations between variables which predicted positive infant faeces. We used Fisher’s exact test as a more appropriate measure for correlation due to the small sample size, which also gives an odds ratio and corresponding p-value, as stated in the text.  We did not perform a regression between variables as it was felt by the team that the sample size was not sufficiently large to perform such an analysis and we felt we would be ‘over-analysing’ the data. As such, a limitation is that the associations here are not part of a model where other factors would be held constant/controlled for, but present associations between exposure variables and the outcome. |
|  | Only the use of microbiology approach (direct plating on selective medium) to identify Campylobacter is not good sufficient enough. Additional molecular approach (PCR) must be performed to confirm the isolates belong to the Campylobacter genus. | We thank you reviewer for their comment and insight here. We have addressed this particular comment above. |
|  | **Introduction:** |  |
|  | The introduction is too long | We thank the reviewer for their comment on the introduction. We politely disagree that the introduction is longer than is usually submitted to PLOS One. We endeavoured to frame the study within current evidence and to address the research need. We will be happy to cut the introduction if further reviews suggest to. |
|  | **Materials and Methods** |  |
|  | Remove text from line 161 to 167. | We thank the reviewer for this suggestion. We respectfully choose to keep the sentence to frame the study within context. The second reviewer also suggests to include more context-specific examples of research within the field. |
|  | Line 173: Replace Zone by zone. Further, what do you mean by zone (=Woreda or kebele)? | Thank you, this is clarified in line 176. |
|  | Line 173-175, More details are required concerning the simple random sampling method. Did the number of free-range chickens per household was recorded? Did the authors saw a correlation between the campylobacter prevalence or other recorded data and the number of free-range chickens. | Thank you. The random sample is already described in the text following (lines 180-188). A line has been added to clarify that the description follows. |
|  | Line 178, “simple lottery method” Can you clarify? | Thank you. The simple lottery method is clarified within the description. |
|  | Line 180-182: I strongly disagree with the authors. The authors used these data to estimate the prevalence of Campylobacter in this specific area in Ethiopia. Therefore, the sampling method and the sample size are crucial in order to properly estimate the prevalence of Campylobacter. Please remove this sentence. Further, the authors must acknowledge that the population size low and restricted to only two kebeles. Please can you provide information concerning the expected statistical power of your analysis based population size. These information must be acknowledge in the manuscript as limitations of your study | We thank the reviewer for their feedback here. We respect the view, however we politely disagree here. Formative research is not intended to test a hypothesis, but is intended as an initial part of the process of a larger study design, and can use both qualitative and quantitative methods to provide data for research teams to plan interventions or further data collection. Formative data is early phase data not powered to detect differences between groups (Gittelsohn et al., 2006).  We have added this description to the methods (lines 190-196) to clarify the positioning of this study. We hope this addresses the reviewer’s concern. |
|  | Line 194-195, please can you explain how the comparison was performed. Did you have reference chart for the consistency (i.e. solid, pasty, liquid)? | Thank you for your comment. The reporting of caregiver-reported diarrhoea is a standard measurement using WHO guidelines, as is referenced in lines 203-206. We have also described in lines 206-208 how we matched with visible diarrhoea. We did not use a chart but diarrhoea was clear from samples as is stated (6 or 7 on the Bristol stool chart). |
|  | Line 202: what is the estimated time between the sampling and the incubation of the inoculated selective plates in microaerophilic conditions? | Thank you, we assume this refers to line 227 and have added a sentence to clarify timing. |
|  | Line 220: did the authors used a positive control in parallel during the processing the field samples (i.e. pure culture of C. jejuni or C. coli for example)? | Thank you. We did not use a positive control within the field. As described in lines 263-266, the media was tested prior to fieldwork using a pure culture and blank samples were used in the field to confirm absence of contamination. |
|  | Line 233: For how long the plates drying? This step is not necessary and might cause the death of Campylobacter. | Thank you for your insight. We left the plates to dry as per manufacturer instructions.  (CHROMagar) before stacking the plates. Indeed other teams reporting similar work in PLOS NTDs stated they left the inoculation to passively diffuse for 30 minutes (Lee G et al. PLoS Negl Trop Dis. (2013), 7(1): e2036).  We left the sample to dry for approximately 5 minutes. This is added in line 252-253. |
|  | Line 233-237: I know by experience that this protocol is not sufficient to isolate Campylobacter from feces in Ethiopia due to the development of dense background (green colonies for example) and/or the presence of red campylobacter-like colonies that are not Campylobacter after confirmation via genus specific PCR (16S primer). | We thank the reviewer for their insight. We are unsure how this sentence aligns with lines 233-237 in the manuscript.  We note in lines 261-263 the prior testing of this Chromagar which shows high sensitivity and specificity. We did see a dense background in some plates when incubated: However, we discussed this with the manufacturer. It was stated that Chromagar media is designed for the isolation of thermotolerant Campylobacter (jejuni, lari, coli) and is very good at suppressing growth of background flora (the enterics such as E.coli and Enterococcus); thus there is confidence that any red colonies on the plate are Campylobacter spp.  Without confirming these results with PCR or other tests, there is little way we can respond: however, we trust in the validity of findings which have shown Chromagar selects for Campylobacter, and have indicated that Chromagar was used for the isolation and **presumptive identification** of thermotolerant Campylobacter (line 240).  To avoid over-claiming on our findings, we use the term ‘presumptive’ where possible in the manuscript.  We would also like to respectfully express we think it is important that these sorts of studies are replicated across different settings and contexts. Where the media may not have been sufficient in one context, that may not be the case across all studies. As with any scientific area, it is important to have more than one set of findings to make a judgement: particularly with new methods (Chromagar) where other authors may replicate protocols and add further evidence. |
|  | Line 241-243: Unfortunately, this statement is not true. Our studies (unpublished data) showed that other genus can grow in this medium, which give campylobacter-like colonies. Due date, the only way we could differentiate them was via aerobic growth test or 16S sequencing. | Thank you for your comment and sharing your experience in the field. We believe we have addressed this comment above.  We would like to stress the same point made above about the importance of sharing findings from similar methodologies in order to strengthen the area of research. |
|  | Line 249-252: I am scared that without PCR confirmation, you cannot affirm that your isolates are from the Campylobacter genus. This limitation must be acknowledged in your manuscript. I am suspecting that you are highly overestimating the Campylobacter prevalence based on your direct plating data. Our team faced the exact same issues in a previous project. Out of 1200 suspected Campylobacter isolates collected in Ethiopia using CHROMagar, less them 5% of the isolates were positive for Campylobacter via PCR. I would recommend buying external generators, which allow to continue your PCR cycles for 3-6 hrs. | We thank the reviewer for this insight and for sharing their experience. We believe we have addressed this comment with our first comment in response to the same comment from them. We agree an external generator would have been great – indeed back-up generators also failed on this occasion. |
|  | Line 273: you do not know whether Campylobacter or another microorganism of the gut microbiota is the causal agent of the diarrhea symptoms observed in the infant; thereby, you cannot use the term infected. It needs to be changed throughout the manuscript. | Apologies we cannot see how this comment refers to line 273.  If the reviewer refers to lines 388 onwards, we have described that this is an association and the relationship with the excretion period of Campylobacter within the stool. We add a line that of course other microorganisms may be present which caused diarrhoeal symptoms (lines 397-398).  Respectfully, we would like to add the point that if we see suspected growth in a stool sample, it is highly likely the infant is infected – Campylobacter is not normally isolated in a stool, and only very small amount of subtypes are part of ‘commensal’ gut flora – most of them are disease-causing. |
|  | **Results:** |  |
|  | Line 311-323: You cannot use the term infected. It needs to be changed throughout the manuscript. All correlation data presented in the manuscript should be associated with a r2 value to fully appreciated the power of the correlation. | Thank you for your comment. As described in the point above, we have described the association. We acknowledge in our paper that Campylobacter is likely not the only cause of infection. We have also altered how we determine ‘infection’ in this paper, as infant stools ‘positive or negative’ for presumptive Campylobacter (lines 288-293). We do not wish to appear we are over-claiming on the data and as such, we have edited both the long and short titles. We hope these are appropriate and reflect the limitations of the small sample size.  We thank the reviewer for their suggestion on the r2 value; we have responded to this in a comment above. |
|  | Line 311: Did you observed difference in Campylobacter prevalence between the two kebeles. Why the authors did not present the CFU data from the direct plating? | Thank you for your comment and questions. We did not separate between the two kebeles. Given the small sample size, we did not believe this would give a meaningful finding. We did not present CFU as this was not the primary aim of the study, we intended to gauge prevalence only. |
|  | Line 341-342: This sentence is for the discussion, not the results | Apologies but we cannot see how lines 341-342 is intended for discussion. |
|  | Line 343-34: This sentence is for the fig legend, not the results | Again apologies but we cannot see how these lines should be removed. We have removed two lines, 360 and 364 if these are those that the reviewer referred to. |
|  | Line 346: the legend in fig 1 and 2 should explain what the p values means. Further the quality of the figures is not good enough (too pixeled). | Thank you for your suggestion. We are unsure exactly what the reviewer means by ‘explain what p value means’ but have added the statement as to the significance boundary to Figure 1. Figure 2 does not contain any p values.  Upon submission to PLOS One the figure file types will be of sufficient quality as requested. |
|  | Line 364: the author must acknowledge the other potential reason of the diarrhea (other microorganism of the microbiota) | Thank you. We think this request refers to the wrong line in the text. We have added this statement in lines 398-399. |
|  | Line 366-368: the population is too small, you cannot do such hypothesis. | Again apologies but we do not think the line the reviewer is referring to corresponds to the manuscript. We have included caveats throughout the manuscript as to limitations with the size of the sample. |
|  | Line 375-377: your data do not support that given you don’t know if campylobacter is the causal agent of the diarrhea. | Thank you. Again we have amended lines 398-399 and is already acknowledged in lines 491-494. |
|  | Line 398-400: Again your population size is too small to make any hypothesis or conclusion. You may suggest a potential transmission from floor to infant but more studies must be done to confirm these preliminary results! | Again we are unsure which line the reviewer refers to. On line 435, we have added ‘potentially’. However many other studies have demonstrated the importance of contaminated floors as an important transmission pathway to infants due to faeces and contamination of the floor surface (including references 27,49,50 on line 137, references 56-58 on line 154 and references 27, 80, 81 on line 445). |
|  | Line 417-419; 453-455; 473-476: same comment then above. Your population size is too small to make any hypothesis or conclusion. These are preliminary results. | Again we are unsure which line the reviewer refers to. In line 481-484 we have stressed the importance that the results are formative and the sample is small. |
|  | The discussion is long and the authors miss to mention anything about the important limitation of this study: the population size and the lack of complete methodology to confirm the nature of the isolates. | Thank you for your comment. We agree the discussion is long; we also included the conclusion. We have removed some paragraphs to reduce the overall length.  We add caveats and limitations throughout the discussion, including:  Lines 154-155  Line 175  Lines 190-196  Lines 481-484  Lines 496-505 |
| **2** | **Abstract:** |  |
|  | Add specific stud area: SNNO has over 14,000,000 population. the sidama population is over 6 million | We thank the reviewer for their comments on the manuscript and we are pleased to send our responses.  Thank you, we have added Sidama zone. |
|  | Add keyword at the end. | Thank you, we have added keywords after the abstract. |
|  | Any association between improved wash and children growth. | Thank you, we think this is already addressed in lines 45-46. |
|  | I do not think there is a author summery after abstract. If not remove it. | Thank you, PLOS One indeed does not require an author summary, this is removed. |
|  | **Introduction:** |  |
|  | Add local studies on prevalence of wash, campylobacter, and stunting in Ethiopia. | Thank you for your suggestion. We have added two studies (line 169-172) and also a further study which was conducted in Hawassa city in the same zone. We have referenced these again in the discussion (lines 393-394). |
|  | **Methods:** |  |
|  | Add detail methods of standardization and cite them | Thank you. We have noted some use of standardized methods in the methodology section. The survey was previously validated (line 199). The Chromagar is used and prepared according to manufacturer’s instructions (line 252). Anthropometry is performed according to WHO guidelines (reference added, line 213). |
|  | Is anthropometry is recommended for children 0-6 months, justify it or include in limitation of the study | Thank you. This study does not include infants under the age of 10 months, for whom anthropometry is appropriate. |
|  | Cite the acceptability of your sample collection and transport methods in low income setting. | Thank you for the suggestion. We have cited some similar research within Ethiopia which used collection and transport methods similar to our study (line 231). |
|  | **Results and discussion:** |  |
|  | I did not found the regression table in the section. | Thank you. As described in the statistical analysis section (lines 301-304), we used the Fisher’s exact test to test associations between variables. Fisher’s exact test is a more appropriate measure for correlation in this study due to the small sample size. The test also gives an odds ratio and corresponding p-value, as stated in the text.  We did not perform a regression between variables as it was felt by the team that the sample size was not large enough. |
|  | Add strength and limitation of this study | Thank you for your comment. As we replied to the first reviewer, we have included caveats and limitations throughout the paper including points about formative research (lines 190-196) and refer to limitations within the discussion, including lines 481-505. |
