# Supplementary material for: Risk factors and transmission pathways associated with infant Campylobacter spp. prevalence and malnutrition: A formative study in rural Ethiopia
Source: PLoS One. 2020 May 8;15(5):e0232541. doi: 10.1371/journal.pone.0232541 (PMC7209302; doi:10.1371/journal.pone.0232541)
Supplement: Attachment — Submitted filename: Reviewer response_060420.docx [file pone.0232541.s006.docx]

| Line | Editor comment | Author comment |
| --- | --- | --- |
| 34 | (N=102) ‘add total’ | We have changed this here and throughout to replace ‘N’ with total |
| 36, 281 | No numeric number at the beginning of a sentence | This has been changed where it occurs |
| 44 | It seems that several place in the manuscript might have double spaces between words. Please check. | We have checked this throughout the manuscript, thank you |
| 146-176 | The majority of the sentence of this section does not belong to the methods section. It seems to be an extension of  the introduction section. | Thank you for the suggestion. We have removed some of the content from the methods section and placed it under the ‘study aims’ section where we feel it fits better. |
| 207 | I do not understand the purpose of this sentence. Clarify or remove it? | This was to demonstrate good biohazard control practices. However we have removed it if it is deemed uncessary. |
| Table 1 | Formating issues. Which column do they belong? | We have moved the average figures into the right column (labelled average or percent), thank you |
