# Supplementary material for: Risk factors and transmission pathways associated with infant Campylobacter spp. prevalence and malnutrition: A formative study in rural Ethiopia
Source: PLoS One. 2020 May 8;15(5):e0232541. doi: 10.1371/journal.pone.0232541 (PMC7209302; doi:10.1371/journal.pone.0232541)
Supplement: Attachment — Submitted filename: reviewer response 150420.docx [file pone.0232541.s008.docx]

Two sentences have been rewritten where previously numbers had been written out in full at the start of the sentence.
